# Altered gene expression in antipsychotic-induced weight gain

**DOI:** 10.1038/s41537-019-0075-y

**Published:** 2019-04-10

**Authors:** Benedicto Crespo-Facorro, Carlos Prieto, Jesus Sainz

**Affiliations:** 1 Department of Psychiatry, School of Medicine, University Hospital Marqués de Valdecilla, IDIVAL, HU Virgen del Rocio-IBIS-Universidad de Sevilla, University of Cantabria, Santander, Spain; 2 CIBERSAM – Centro Investigación Biomédica en Red Salud Mental, Santander, Spain; 3 Bioinformatics Service, Nucleus, University of Salamanca (USAL), Salamanca, Spain; 4 Spanish National Research Council (CSIC), Institute of Biomedicine and Biotechnology of Cantabria (IBBTEC), Santander, Spain

## Abstract

Antipsychotic drugs are one of the largest types of prescribed drugs. However, antipsychotic-induced weight gain (AIWG) is a major problem for the patients. AIWG increases cardiovascular and cerebrovascular morbidity and mortality, and reduces quality of life and drug compliance. To characterize changes in gene expression related to AIWG, we sequenced total messenger RNA from the blood samples of two groups of schizophrenia patients before and after 3 months of treatment with antipsychotics. The “weight gain” group was defined by an increase of body mass index (BMI) >1.5 points (18 patients; median BMI increase = 2.69) and the “no weight gain” group was defined by a change of BMI between <1.0 and >−1.0 points (18 patients; median BMI increase = 0.26). We found 115 genes with significant differential expression in the weight gain group before and after medication and 156 in the no weight gain group before and after medication. The weight gain group was significantly enriched with genes related to “obesity” and “BMI” (Fisher; *p* = 0.0002 and 0.01, respectively) according to the Gene Reference into Function (GeneRIF) database. In the no weight gain group, the enrichment was much smaller (Fisher; *p* = 0.02 and 0.79). This study is a first step toward detecting genetic factors that cause AIWG and to generating prediction tests in future studies with larger data sets.

## Introduction

Antipsychotic drugs are widely used to treat mental health problems including schizophrenia,^1,2^ bipolar disorder, depression, dementia, and autism among other clinical conditions.^3–5^ They are one of the most prescribed and costly groups of drugs in clinical practice.^6^ Despite their frequent use, these types of drugs are significantly associated with weight gain.^7–9^ Weight gain affects compliance with the treatment and is indirectly responsible for psychosis relapses.^10^ Additionally, weight gain is a risk factor for type II diabetes and can lead to metabolic syndrome.^11^ The consequences of long-term treatment can also result in an increased risk for cardiovascular diseases.^12–14^ Overall, patients with schizophrenia have a significantly reduced life expectancy, compared to the general population.^15–18^ Remarkable differences in individuals with antipsychotic-induced weight gain (AIWG) suggest a strong genetic component.^19^ To characterize the genetic component of AIWG, we studied the gene expression related to the weight gain caused by antipsychotics.

We sequenced the transcriptome of two cohorts of first-episode schizophrenia patients before and after 3 months of treatment with antipsychotics (Table 1). The transcriptomes of each group were analyzed independently to define the genes with significant differential expression before and after medication using the program Deseq.^20^ The first cohort, or the “weight gain” group, included 18 individuals who gained more than 1.5 points of BMI after the treatment. The second cohort, the “no weight gain” group, also included 18 individuals with a change in BMI after the treatment between 1.0 and −1.0 points.

**Table 1:** Anthropometric and metabolic values in both groups of individuals studied

Median	Weight gain			No weight gain		
	All (*N* *=* 18)	Male (*N* *=* 9)	Female (*N* *=* 9)	All (*N* *=* 18)	Male (*N* *=* 9)	Female (*N* *=* 9)
Age (years)	25.1	26.2	23.2	29.8	39.8	25.6
BMI before medication	21.7	22.1	21.5	20.8	26.8	19.9
BMI after medication	24.7	24.8	23.7	21.2	26.2	20.4
BMI increase	2.7	2.7	2.5	0.3	0.3	0.2
Triglycerides before medication (mg/dl)	64.5	85.0	62.0	65.0	75.0	58.5
Triglycerides after medication (mg/dl)	102.0	107.0	87.0	76.0	76.0	66.0

There are 155 genes with significant differential expression between the weight gain and no weight gain groups before drug treatment. The weight gain group has 115 genes with significant differential expression before and after 3 months of medication. The no weight gain group has 156 genes with significant differential expression before and after 3 months of medication. There are 301 genes with significant differential expression between the weight gain and no weight gain groups after 3 months of drug treatment.

## Results

### Differential gene expression between the “weight gain” and the “no weight gain” groups before treatment with antipsychotics

We found 155 genes with significant differential expression between the weight gain and no weight gain groups before drug treatment (*p*_adj_ value < 0.01) (Supplementary Table S1; Table 2 contains the most significant genes, *p*_adj_ value < 1.0E20). In all analyses, to characterize the functionality of these genes, we used the Gene Reference into Function (GeneRIF) database.^21^ We considered “obesity-,” “BMI-,” “cholesterol-,” or “trigliceride-” related genes to be those that contain the string “obesity,” “BMI,” “cholest,” or “trigly,” respectively, in the GeneRIF concise phrase that describes the function or functions of the gene.

**Table 2:** Most significant differential expression genes between the weight and the no weight gain groups before medication

Gene symbol	Base mean weight gain	Base mean no weight gain	Log 2 fold change	*P*_adj_	GeneRif annotation
*HBG1*	184.32	1282.13	2.80	1.22E−83	CHOLEST
*OTOF*	213.17	55.78	−1.93	7.46E−62	
*IFI44L*	3789.40	1598.25	−1.25	3.93E−28	
*SIGLEC1*	2042.72	943.07	−1.12	6.56E−26	
*RSAD2*	2611.27	1021.60	−1.35	2.85E−25	
*C21orf15*	399.10	165.24	−1.27	6.80E−23	
*HBG2*	1364.10	3524.10	1.37	1.54E−21	CHOLEST
*NEBL*	93.49	272.09	1.54	2.32E−21	

Gene symbol: official symbol; base mean weight gain: mean normalized counts from condition A; base mean no weight gain: mean normalized counts from condition B; base mean no weight gain: mean normalized counts from condition B; log 2 fold change: the logarithm, to basis 2, of the fold change; *P*_adj_: *p* value adjusted for multiple testing with the Benjamini–Hochberg procedure, which controls false discovery rate; GeneRif annotation: genes including the strings “BMI” or “cholest” in their GeneRIF definition

Using the previous criteria, the 155 genes with differential gene expression between the “weight gain” and the “no weight gain” groups, before treatment with antipsychotics, were not significantly enriched for “obesity-,” “BMI-,” “cholesterol-,” or “triglyceride-” related genes. We found eight obesity-related genes or 6.1% of the annotated genes, while we expected 5.1% (Fisher; *p* = 0.0544). When we analyzed for the string BMI, we found four genes or 3.1% of the annotated genes, while we expected 2.9% (Fisher; *p* = 0.7903). The “cholesterol-” related genes were eight genes or 6.2% of the annotated genes, while we expected 5.3% (Fisher; *p* = 0.6924). The “triglyceride-” related genes were three genes or 2.3% of the annotated genes, while we expected 1.9% (Fisher; *p* = 0.7390)

### Differential gene expression in the “weight gain” group before and after treatment with antipsychotics

In the weight gain patients, we found 115 genes with significant differential expression before and after 3 months of medication (*p*_adj_ value < 0.01) (Supplementary Table S2; Table 3 contains the most significant genes, *p*_adj_ value < 1.0E20).

**Table 3:** Most significant differential expression genes in the weight gain group before and after 3 months of medication

Gene symbol	Base mean before medication	Base mean after medication	Log 2 fold change	*P*_adj_	GeneRif annotation
*MTRNR2L2*	24.81	494.49	4.32	3.27E−135	
*IFI27*	127.06	24.68	−2.36	1.56E−51	
*OTOF*	193.19	60.32	−1.68	2.34E−45	
*LOC100190986*	8.81	89.54	3.35	8.45E−38	
*OLFM4*	217.58	605.17	1.48	7.53E−34	
*ADAMTS2*	72.83	13.38	−2.44	3.68E−33	
*LTF*	2039.45	5013.98	1.30	1.55E−31	
*LCN2*	566.25	1383.66	1.29	1.03E−30	BMI
*MMP8*	297.24	695.90	1.23	6.80E−27	CHOLEST
*ABCA13*	155.82	399.40	1.36	9.71E−27	
*CEACAM8*	321.14	792.53	1.30	3.47E−26	

Gene symbol: official symbol; base mean before medication: mean normalized counts from condition A; base mean after medication: mean normalized counts from condition B; log 2 fold change: the logarithm, to basis 2, of the fold change; *P*_adj_: *p* value adjusted for multiple testing with the Benjamini–Hochberg procedure, which controls false discovery rate; GeneRif annotation: genes including the strings “BMI” or “cholest” in their GeneRIF definition

Using the criteria defined in the previous section, these genes were significantly enriched for “obesity-,” “BMI-,” “cholesterol-,” or “triglyceride-” related genes according to the scientific literature in the GeneRIF databases.^21^

We found 15 “obesity-” related genes with differential expression or 14.7% of the annotated genes, while we expected 5.1% (Fisher; *p* = 0.0002). When we analyzed for BMI, we found eight genes or 7.8% of the annotated genes, while we expected 2.9% (Fisher; *p* = 0.0097). The “cholesterol-” related genes were 13 or 12.7% of the annotated genes, while we expected 5.1% (Fisher; *p* = 0.003). The “triglyceride-” related genes were 6 or 5.9% of the annotated genes, while we expected 1.9% (Fisher; *p* = 0.014).

### Differential gene expression in the “no weight gain” group before and after treatment with antipsychotics

In the no weight gain patients, we found 156 genes with significant differential expression before and after 3 months of medication (*p*_adj_ value < 0.01) (Supplementary Table S3; Table 4 contains the most significant genes, *p*_adj_ value < 1.0E20).

**Table 4:** Most significant genes with differential expression in the no weight gain group before and after 3 months of medication

Gene symbol	Base mean before medication	Base mean after medication	Log 2 fold change	*P*_adj_	GeneRif annotation
*MTRNR2L2*	1108.97	41.38	5.37	4.57E−222	
*LOC100190986*	112.72	11.74	3.55	3.05E−48	
*IDO1*	592.70	2.39	1.26	7.77E−36	BMI
*ANKRD22*	437.44	2.34	1.23	7.92E−28	
*ADAMTS2*	20.24	0.24	−2.06	2.71E−27	
*CXCL10*	208.90	2.54	1.34	2.47E−26	
*ART3*	223.32	2.47	1.30	5.61E−26	
*DHFR*	1808.97	2.57	1.36	4.56E−22	
*GBP5*	19955.17	1.90	0.93	2.58E−21	
*SERPING1*	1107.86	1.93	0.95	5.52E−21	

Gene symbol: official symbol; base mean before medication: mean normalized counts from condition A; base mean after medication: mean normalized counts from condition B; log 2 fold change: the logarithm, to basis 2, of the fold change; *P*_adj_: *p* value adjusted for multiple testing with the Benjamini–Hochberg procedure, which controls false discovery rate; GeneRif annotation: genes including the strings “BMI” or “cholest” in their GeneRIF definition

These genes were not enriched for “BMI”, “cholesterol,” or “triglyceride”, and had a weak enrichment for “obesity-” related genes according to the GeneRIF database.^21^

We found 13 “obesity-” related genes with differential expression or 9.8% of the annotated genes, while we expected 5.3% (Fisher; *p* = 0.026). When we analyzed for the string BMI, we found four genes or 3.0% of the annotated genes, while we expected 2.9% (Fisher; *p* = 0.79).

The differential expression genes were not significantly enriched for “cholesterol-” related genes: nine genes or 6.8% of the annotated genes, while we expected 5.3% (Fisher; *p* = 0.44), or for “triglyceride-” related genes: three genes or 2.3% of the annotated genes, while we expected 1.9% (Fisher; *p* = 0.74)

**Fig. 1:**
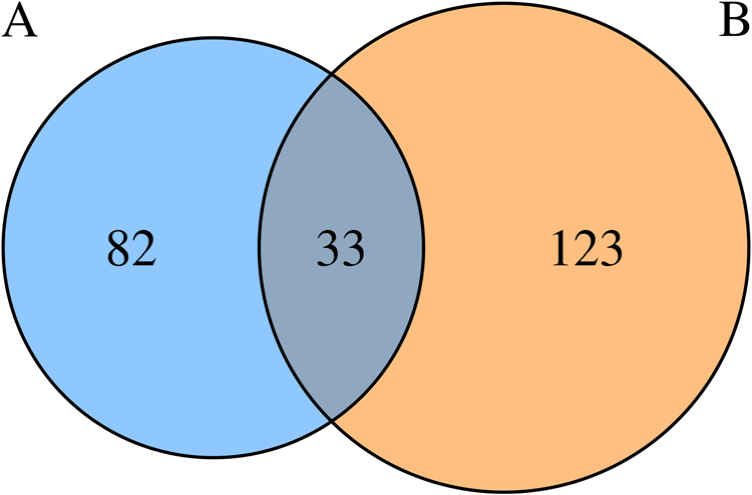
Number of genes with expression altered by antipsychotics. **a** “Weight gain” group before and after antipsychotic medication. **b** “No weight gain” group before and after antipsychotic medication

Comparing the differentially expressed genes before and after the antipsychotic treatment of the weight gain individuals vs. the no weight gain individuals, we found 33 common genes (Fig. 1). There were 82 differentially expressed genes present in the weight gain group, but not in the no weight gain group (Fig. 1). Twelve of the 82 genes only in the weight gain group were “obesity” related and seven were “BMI” related according to the GeneRIF database. Common to both groups were three obesity genes and one BMI gene, and these genes had their expression altered by the drugs in the same direction in both groups, up- or downregulated (Supplementary Tables S2 and S3).

### Differential gene expression between the “weight gain” and the “no weight gain” groups after treatment with antipsychotics

**Fig. 2:**
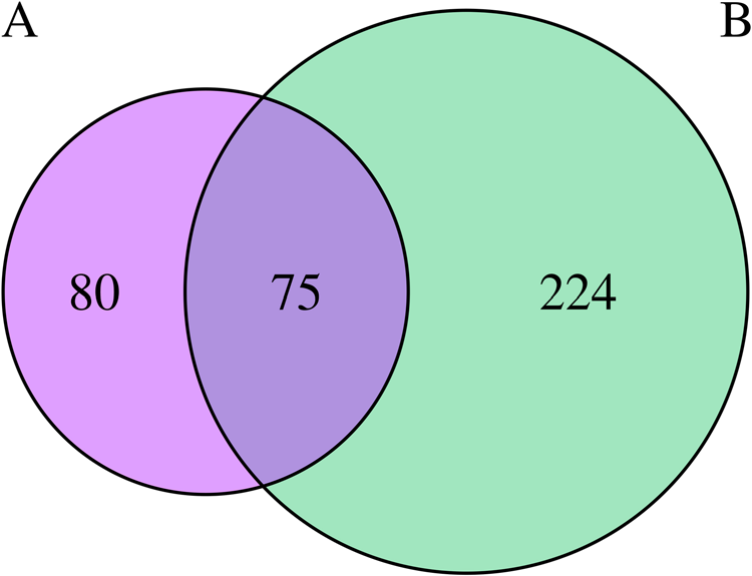
Number of genes with differential expression between the weight and no weight gain groups. **a** Before antipsychotic medication. **b** After antipsychotic medication

We found 301 genes with significant differential expression between the weight gain and no weight gain groups after 3 months of drug treatment (*p*_adj_ value < 0.01) (Supplementary Table S4; Table 5 contains the most significant genes, *p*_adj_ value < 1.0E20). Seventy-five out of the 301 genes also had differential expression between both groups before medication (Fig. 2). According to GeneRIF data, these 301 genes were significantly enriched for “obesity-” and “cholesterol-” related genes and had a weak enrichment for “triglyceride-” related genes. There was no enrichment for “BMI-” related genes.

**Table 5:** Most significant differential expression genes between weight and no weight gain groups after medication

Gene symbol	Base mean weight gain	Base mean no weight gain	Log 2 fold change	*P*_adj_	GeneRif annotation
*HBG1*	145.94	622.58	2.09	1.42E−73	CHOLEST
*MTRNR2L2*	486.77	1001.34	1.04	3.70E−47	
*PRAME*	29.23	2.13	−3.78	1.29E−25	
*MMP8*	683.11	296.46	−1.20	5.05E−24	CHOLEST
*CPA5*	69.68	16.13	−2.11	5.73E−23	
*ETV7*	167.26	372.19	1.15	6.64E−23	
*ANKRD22*	191.43	395.08	1.05	6.50E−21	

Gene symbol: official symbol; base mean weight gain: mean normalized counts from condition A; base mean no weight gain: mean normalized counts from condition B; log 2 fold change: the logarithm, to basis 2, of the fold change; *P*_adj_: *p* value adjusted for multiple testing with the Benjamini–Hochberg procedure, which controls false discovery rate; GeneRif Annotation: Genes including the strings “BMI” or “cholest” in their GeneRIF definition

We found 25 “obesity-” related genes or 10.0% of the annotated genes, while we expected 5.1% (Fisher; *p* = 0.0013). The “cholesterol-” related genes were 25 or 10.0% of the annotated genes, while we expected 5.3% (Fisher; *p* = 0.0028). The “triglyceride-” related genes were 10 or 4.0% of the annotated genes, while we expected 1.9% (Fisher; *p* = 0.0307). We found nine “BMI-” related genes or 3.6% of the annotated genes, while we expected 2.9% (Fisher; *p* = 0.4451).

### Pathway analyses

We analyzed several pathway databases (Reactome,^22^ KEGG,^23^ WikiPathways,^24^ and Pathway Interaction Database^25^) to detect the enrichment with differentially expressed genes. Below, we provide information about the four most significantly enriched pathways (the four pathways with the lowest *p* values) in each one of the analyses. Table 6 contains all the genes from these four most significant pathways in each on the four analyses performed.

**Table 6:** Differential expression genes included in the four most significant pathways of all four analyses

Analysis	Pathway	Repository	Gene symbols
Weight gain vs. no weight gain before medication	Interferon signaling	Reactome	*IFI6, FCGR1A, IFIT3, IFIT1, IFITM3, OAS3, IFI27, XAF1, RSAD2, HLA-DRB5, HLA-DRB1, HLA-DQB1*
Weight gain vs. no weight gain before medication	Interferon α/β signaling	Reactome	*IFI6, IFIT3, IFIT1, IFITM3, OAS3, IFI27, XAF1, RSAD2*
Weight gain vs. no weight gain before medication	Immune system	Reactome	*C1QC, IFI6, FCGR1A, IFIT3, IFIT1, IFITM3, OAS3, OLFM4, IFI27, TNFRSF17, XAF1, TNFRSF13B, ITGA2B, ITGB3, CD79A, CD177, SIGLEC11, SIGLEC8, LAIR2, RSAD2, TNFAIP6, SIGLEC1, PI3, LAMA5, PROS1, MUC20, PPBP, DSP, TXNDC5, HLA-DRB5, HLA-DRB1, HLA-DQB1, NEFL, CD274, LCN2*
Weight gain vs. no weight gain before medication	Cytokine signaling in immune system	Reactome	*IFI6, FCGR1A, IFIT3, IFIT1, IFITM3, OAS3, IFI27, TNFRSF17, XAF1, TNFRSF13B, ITGA2B, ITGB3, RSAD2, LAMA5, HLA-DRB5, HLA-DRB1, HLA-DQB1, NEFL, LCN2*
Weight gain before and after medication	Neutrophil degranulation	Reactome	*S100A9, CHIT1, TCN1, MS4A3, MMP8, OLR1, OLFM4, RNASE3, CTSG, MPO, AZU1, PRTN3, ELANE, C19orf59, CEACAM6, CEACAM8, CD177, LILRA3, TNFAIP6, BPI, LTF, CAMP, CRISP3, ABCA13, MGAM, DEFA4, DEFA1, DEFA1B, LCN2*
Weight gain before and after medication	Innate immune system	Reactome	*C1QA, C1QC, C1QB, S100A9, AIM2, CHIT1, C4BPA, MUC2, TCN1, MS4A3, MMP8, OLR1, OLFM4, RNASE3, CTSG, MPO, AZU1, PRTN3, ELANE, C19orf59, CEACAM6, CEACAM8, CD177, LILRA3, TNFAIP6, BPI, LTF, CAMP, C2, CRISP3, ABCA13, MGAM, DEFA4, DEFA1, DEFA1B, DEFA3, LCN2*
Weight gain before and after medication	Immune system	Reactome	*C1QA, C1QC, C1QB, S100A9, AIM2, CHIT1, C4BPA, IFITM3, MUC2, TCN1, MS4A3, MMP8, OLR1, FLT3, OLFM4, RNASE3, CTSG, IFI27, ALOX15, MPO, AZU1, PRTN3, ELANE, C19orf59, CEACAM6, CEACAM8, CD177, PVRL2, SIGLEC8, LILRA3, LILRA5, RSAD2, TNFAIP6, SIGLEC1, BPI, LTF, CAMP, C2, CRISP3, ABCA13, MGAM, DEFA4, DEFA1, DEFA1B, DEFA3, LCN2*
Weight gain before and after medication	Antimicrobial peptides	Reactome	*S100A9, RNASE3, CTSG, PRTN3, ELANE, BPI, LTF, CAMP, DEFA4, DEFA1, DEFA1B, DEFA3, LCN2*
No weight gain before and after medication	Glucuronidation	WikiPathways	*UGT1A8, UGT1A10, UGT1A9, UGT1A7, UGT1A6, UGT1A3, UGT1A1, UGT2B11, UGT2B28*
No weight gain before and after medication	Ascorbate and aldarate metabolism	KEGG	*UGT1A8, UGT1A10, UGT1A9, UGT1A7, UGT1A6, UGT1A3, UGT1A1, UGT2B11, UGT2B28*
No weight gain before and after medication	Glucuronate pathway (uronate pathway)	KEGG	*UGT1A8, UGT1A10, UGT1A9, UGT1A7, UGT1A6, UGT1A3, UGT1A1, UGT2B11, UGT2B28*
No weight gain before and after medication	Pentose and glucuronate interconversions	KEGG	*UGT1A8, UGT1A10, UGT1A9, UGT1A7, UGT1A6, UGT1A3, UGT1A1, UGT2B11, UGT2B28*
Weight gain vs. no weight gain after medication	Neutrophil degranulation	Reactome	*CHIT1, MS4A3, MMP8, OLR1, KRT1, OLFM4, RNASE3, CTSG, HP, MPO, SERPINB10, AZU1, PRTN3, ELANE, CEACAM6, CEACAM8, CD177, PGLYRP1, BPI, SLPI, MMP9, LTF, CAMP, PPBP, DSP, CRISP3, ARG1, ABCA13, CLEC5A, DEFA4, DEFA1, DEFA1B, LCN2*
Weight gain vs. no weight gain after medication	Immune system	Reactome	*C1QB, GBP1, GBP4, GBP5, CD101, SLAMF7, SH2D1B, CHIT1, MS4A3, MMP8, NCAM1, KLRF1, OLR1, KLRD1, KLRC2, KLRC1, KRT1, OLFM4, RNASE3, CTSG, HP, CCL4, ITGA2B, ITGB3, MPO, SERPINB10, AZU1, PRTN3, ELANE, MUC16, CEACAM6, CEACAM8, CD177, PVRL2, PGLYRP1, SIGLEC11, SIGLEC6, LILRA4, KIR2DL3, KIR2DL1, KIR2DL4, KIR3DL1, KIR2DS4, KIR3DL2, NCR1, GNLY, DUSP2, BPI, PI3, SLPI, MMP9, LTF, CAMP, PROS1, PPBP, CXCL10, IL31RA, ERAP2, PDGFRB, DSP, HLA-DRB5, HLA-DRB1, HLA-DQB1, HLA-DQB2, TREML4, CRISP3, ARG1, ABCA13, COL1A2, CLEC5A, DEFA4, DEFA1, DEFA1B, DEFA3, LCN2*
Weight gain vs. no weight gain after medication	Graft-vs.-host disease	KEGG	*PRF1, KLRD1, KLRC1, GZMB, KIR2DL3, KIR2DL1, KIR3DL1, KIR3DL2, HLA-DRB5, HLA-DRB1, HLA-DQB1*
Weight gain vs. no weight gain after medication	Immunoregulatory interactions between a lymphoid and a non-lymphoid cell	Reactome	*SLAMF7, SH2D1B, KLRF1, KLRD1, KLRC1, PVRL2, SIGLEC11, SIGLEC6, LILRA4, KIR2DL3, KIR2DL1, KIR2DL4, KIR3DL1, KIR3DL2, NCR1, TREML4*

*KEGG* Kyoto Encyclopedia of Genes and Genomes

The differentially expressed genes between the weight gain and no weight gain groups before medication were enriched mainly in pathways related to the immune system. The four most significantly enriched pathways were all from Reactome. For the interferon signaling pathway, we found 12 genes (observed genes 13%, expected 2%; Fisher *p* = 4.9E−08). For the interferon α/β signaling pathway, we found eight genes (observed genes 9%, expected 1%; Fisher *p* = 1.0E−07). For the immune system pathway, we found 35 genes (observed genes 38%, expected 18%; Fisher *p* = 2.8E−06). For the cytokine signaling in immune system pathway, we found 19 genes (observed genes 21%, expected 6%; Fisher *p* = 4.0E−06)

In the weight gain group before and after medication, the four most significantly enriched pathways were immune system related (all from Reactome). For the neutrophil degranulation pathway, we found 29 genes (observed genes 33%, expected 4%; Fisher *p* = 1.5E−18). For the innate immune system pathway, we found 37 genes (observed genes 42%, expected 11%; Fisher *p* = 8.1E−14). For the immune system pathway, we found 46 genes (observed genes 52%, expected 18%; Fisher *p* = 2.4E−13). For the antimicrobial peptides pathway, we found 13 genes (observed genes 15%, expected 1%; Fisher *p* = 5.5E−12). Eighty-eight out of the 115 differentially expressed genes in the weight gain group before and after drug treatment were pathway annotated. The majority of these genes, 46 genes (52% of the 88), were included in the four most significant pathways mentioned above.

The no weight gain group did not show such strong enrichment of differentially expressed genes for immune system-related pathways. The four pathways more significantly enriched were the following. Glucuronidation (WikiPathways), with nine genes (observed genes 8%, expected 0.23%; Fisher *p* = 3.3E−11). Ascorbate and aldarate metabolism (KEGG) and glucuronate pathway (uronate pathway) (KEGG) both of them with nine genes (observed genes 8%, expected 0.24%; Fisher *p* = 4.4E−11), and pentose and glucuronate interconversions (KEGG) with nine genes (observed genes 8%, expected 0.30%; Fisher *p* = 2.4E−10).

The differentially expressed genes of the weight gain and no weight gain group after medication were enriched most significantly in the four following pathways: neutrophil degranulation (Reactome) revealed 33 genes (observed genes 17%, expected 4%; Fisher *p* = 1.6E−11); the immune system pathway (Reactome), with 75 genes (observed genes 38%, expected 18%; Fisher *p* = 3.1E−11); graft-vs.-host disease (KEGG), with 11 genes (observed genes 6%, expected 0.3%; Fisher *p* = 2.4E−10); and immunoregulatory interactions between a lymphoid and a non-lymphoid cell (Reactome) with 16 genes (observed genes 8%, expected 1%; Fisher *p* = 4.7E−10).

## Discussion

It is known that antipsychotics induce weight gain^10^ and that genetic factors play a major role in this weight gain.^8,26^ Our results characterized 115 genes with expression significantly altered by the antipsychotics after 3 months of medication in a “weight gain” group of individuals who increased their BMI >1.5 after the treatment (median BMI gain = 2.7). Excluding the genes also altered by medication in the no weight gain group (individuals with small changes of BMI, <1.0 and >−1.0; median BMI change = 0.3), we characterized 82 genes with expression significantly altered by antipsychotics only in the weight gain group (Fig. 1). Twenty-one of these 82 genes have been associated with obesity, obesity-related traits or metabolic syndrome in the scientific literature. Thirteen of these 21 genes (*LTF*,^27^ *LCN2*,^28^ *MMP8*,^29^ *OLR1*,^30^ *MPO*,^31^ *ABCA1*,^32^ *SDC3*,^33^ *MSR1*,^34^ *H19*,^35^ *FABP1*,^36^ *ADM*,^37^ *S100A9*,^38^ and *CHIT1*^39^) appear in functional studies in the GeneRIF database.^21^ Ten of the 21 genes (*PDE3A*,^40^ *ABCA1*,^41^ *FPR3*,^40^ *MYO16*,^42^ *LILRA3*,^40^ *RNASE1*,^42^ *ADM*,^43^ *KYNU*,^44^ *AIM2*,^42^ and *LILRA5*^40^) appear in association studies according to the genome-wide association studies (GWAS) catalog,^45^ with two of these genes (*ABCA1* and *ADM*) common to GWAS and GeneRIF. Interestingly, *ADM* has been associated with blood lipid levels^43^ and with bipolar disorder^46^ and is a gene that promotes antimicrobial activity.^47^ Another three genes, in addition to obesity-related traits, also associate with psychotic disorders, such as schizophrenia (*MYO16*^48^ and *ABCA1*^49^) or bipolar disorder (*FPR3*^50^). *ADM* and *FPR3* appear in several common Reactome pathways (G protein-coupled receptor (GPCR) downstream signaling, GPCR ligand binding, signal transduction, and signaling by GPCR), suggesting a similar mechanism of action. These data indicate links between psychotic disorders, obesity, and the immune system.

When we searched for “obesity” in the GeneRIF database, we found a significant enrichment of genes with altered expression after antipsychotic medication in the weight gain group (Fisher; *p* = 0.0002). We found 82 genes with expression altered by antipsychotics in the weight gain group, but not altered in the no weight gain group (Fig. 1). Given that these 82 genes contain a significant enrichment of obesity genes, it is likely that they will also contain novel obesity genes.

In the weight gain group, we found a very significant enrichment of genes with an expression altered by antipsychotics in pathways from the immune system. Forty-six out of the 88 annotated genes (52% of the total) belong to immune system pathways. Among these 46 immune system genes, 12 of them (or 26%) were previously associated with obesity-related traits and 10 of them had expression altered by antipsychotics only in the weight gain group (*LTF*, *LCN2*, *MMP8*, *OLR1*, *MPO*, *LILRA3*, *AIM2*, *LILRA5*, *S100A9*, and *CHIT1*). Foreign pathogens activate the immune system to provide protection against them. Given that metabolic diseases may also activate the immune system, adipose tissue is considered not only a storage of fat but also an endocrine organ.^51^ Obesity alters immune functions and alters leukocyte counts as well as immune responses,^52^ and is a state of chronic low-grade inflammation and a disturbance of metabolic homeostasis that leads to aberrant immune responses.^53–55^ Considering that there is an inflammatory response that appears in the presence of obesity,^53,56,57^ we could expect, as we observed, that AIWG would result in an alteration of the immune system gene expression. We need to consider also that the limitation of using blood samples for the study, rather than samples from the brain, may explain the heavy load of “immune-” related genes.

When we analyzed the 155 differentially expressed genes between the weight group and no weight gain groups before drug treatment, we did not find any significant enrichment for genes related to obesity, BMI, cholesterol, or triglycerides. However, some of these 155 genes could have the potential to predict AIWG that we were unable to find.

Our results indicate that the genes with expression altered by antipsychotics in individuals who gain weight include a very high portion of immune system genes (52%). This observation supports previous studies indicating that lipids regulate metabolic and immune processes in a coordinated manner.^58^ However, in our analyses the vast majority of pathways significantly enriched with genes with an altered expression by antipsychotics in the weight gain group were immune system related but not metabolism related.

Several variables such as age,^16,59–61^ sex,^12^ and type of antipsychotic^10,17^ affect the extent of AIWG. We were able to minimize a bias in sex and type of antipsychotic between the two groups analyzed in this study. The number of individuals taking each type of antipsychotic (nine were medicated with Aripiprazole and nine with Risperidone) were the same in both groups. We also had the same number of males and females (nine) in each group (Table 1). However, we observed that the weight gain group had a median age of 4.7 years lower than the no weight gain group, suggesting that younger people are more sensitive to AIWG.

We know that underlying genetic variation can strongly affect the antipsychotic effect to alter gene expression in different individuals according to their genetic profile. However, given the small sample number available, we were not able to analyze these possible effects.

In conclusion, our study provides functional data on genes that are good candidates for AIWG. Moreover, our study provides additional evidence of the genetic links between weight gain and the immune system. Finally, we consider this study a preliminary step for future studies to confirm the results with a larger group of patients and with the power to design tests to predict weight gain in patients before antipsychotic medication.

## Materials and methods

### Ethics

Conforming to the international standards for research ethics, this study was approved by the Cantabria Ethics Institutional Review Board (IRB).

### Study setting and subjects

The cohort analyzed in this study was obtained from patients who met the following criteria: (1) 15–60 years old; (2) living in the catchment area (Cantabria); (3) experiencing a first episode of psychosis; (4) having received no prior treatment with antipsychotic medications; and (5) Diagnostic and Statistical Manual of Mental Disorders, 4th Edition (DSM-IV) criteria for schizophrenia, schizophreniform disorder, schizoaffective disorder, or brief psychotic disorder. Patients were excluded for any of the following reasons: (1) meeting the DSM-IV criteria for drug dependence, (2) meeting the DSM-IV criteria for mental retardation, or (3) having a history of neurological disease or head injury. The diagnoses were confirmed using the Structured Clinical Interview for DSM-IV (SCID-I) carried out by an experienced psychiatrist 6 months from the baseline visit. Our operational definition for a “first episode of psychosis” includes individuals with non-affective psychosis (meeting the inclusion criteria defined above) who have not previously received antipsychotic treatment regardless of the duration of psychosis. Patients had to meet the inclusion criteria and provide written informed consent to be included in the study. Before giving consent, patients were evaluated by a clinician to assess their competency (their capacity of understanding, reasoning, and expression of a choice was evaluated). The biological samples of patients were provided by the Valdecilla biobank. All methods were performed in accordance with the relevant guidelines and regulations. Individuals who gave written consent for their participation in the program, who fulfilled the inclusion criteria at 6 months, and who had messenger RNA (mRNA) samples at baseline and at 3 months, were included in our analyses.

The adherence to antipsychotic drugs was assessed by the information thoroughly obtained from the patients (self-reported based on the four-item Morisky Green Levine Medication Adherence Scale), their close relatives, and by the staff (nurses, psychologists, social workers, and psychiatrists) involved in the follow-up. Patients were consensually dichotomized into having a good (defined as patients regularly taking at least 90% of the prescribed medication) or poor adherence (medium or poor compliance). Only individuals considered as good adherence to antipsychotic treatment during the 3-month follow-up were included in the present investigation.

Anthropometric and metabolic values were collected from the patients (Table 1).

### Sample study

We sequenced mRNA from blood samples before and after 3 months of treatment with antipsychotics. We defined the “weight gain” group by an increase of BMI >1.5 points (18 patients); and the “no weight gain” group by a change of BMI between <1.0 and >−1.0 points (18 patients).

No significant differences in cumulative dose of antipsychotics during the 3 months, estimated by chlorpromazine equivalent doses,^62^ were observed between the groups (weight gain group 401.91 ± 203.14 mg/day; no weight gain group 337.75 ± 104.25mg/day; *p* = 0.241). At baseline, patients were equally distributed either on risperidone or on aripiprazole (weight gain: *N* *=* 9 on aripiprazole and *N* *=* 9 on risperidone; no weight gain: *N* *=* 9 on aripiprazole and *N* *=* 9 on risperidone). No differences between groups were found in the number of patients taking antidepressants during the study period (*N* *=* 3, 16.7%, patients in the weight gain group and *N* *=* 2, 11.1%, in the no weight gain group; *p* = 0.630).

### Laboratory assessments

Blood samples were assessed for biochemical parameters (glucose and lipid metabolism parameters). To minimize the effects of diet and technique, blood samples were obtained from fasting subjects from 8:00 to 10:00 a.m. by the same staff, in the same setting. Patients were asked to abstain from all food and drinks except water for 8 to 10 h beforehand. A detailed description of the methodology followed to assess the biochemical variables is available upon request. None of the patients had chronic inflammation, infection, or were taking medication that could influence the results of blood tests.

### RNA extraction

Total RNA was extracted from blood using the Tempus™ Blood RNA Tube and the Tempus™ Spin RNA Isolation Kit (Applied Biosystems, Foster City, CA, USA) following the manufacturer's protocols. To define expression profiles, a key factor is that the RNA is intact. To select only high-quality RNA, the RNA integrity number (RIN) was characterized with a Bioanalyzer (Agilent Technologies, Santa Clara, CA, USA) and samples with a RIN of at least 7.6 were used. The selected samples had RINs that range from 7.6 to 10 with an average of 9.11.

### RNA next-generation sequencing

Total RNA was extracted from peripheral blood of each individual. The mRNA obtained from blood was sequenced at the Centro Nacional de Análisis Genómico (CNAG) using Illumina HiSeq instruments (San Diego, CA, USA). The mRNA was isolated from the total RNA and was fragmented once transformed into complementary DNA (DNA). Fragments of 300bp on average were selected to construct the cDNA libraries for sequencing. Pair-end sequences of 70 nucleotides for each end were produced.

### Alignment of reads to the human genome reference

Alignment of the reads was performed in an SLURM HPC server running Tophat 2.0.6 with default options.^63^ Tophat aligns RNA-seq reads to genomes using the Bowtie 2.0.2 alignment program,^64^ and then analyzes the mapping results to identify splice junctions between exons.

### Differential expression statistical analysis

Bedtools 2.17.0 (multicov option)^65^ was used to count the number of reads mapped to each gene. The Reference Sequence (RefSeq) gene coordinates were defined using the RefFlat file from the UCSC Genome Bioinformatics Site (February 28, 2014). The Deseq 1.4 package,^20^ setting up fit-only as the fitting method, was used to test for differential expression using gene-count data. *P* value was adjusted for multiple testing with the Benjamini–Hochberg procedure, which controls false discovery rate. Two-sided Fisher's tests were carried out to identify functional enrichment of biological annotations.

### Reporting Summary

Further information on experimental design is available in the Nature Research Reporting Summary linked to this article.

## Supplementary information

Supplementary Tables S1-S4.

Reporting Summary

## Data Availability

The data that support the findings of this study are available upon request from the corresponding authors. RNA sequences are deposited at the European Nucleotide Archive (ENA) with accession number PRJEB31532.

## Supplementary information

**Supplementary information** accompanies the paper on the *npj Schizophrenia* website (10.1038/s41537-019-0075-y).
