# Supplementary material for: Altered gene expression in antipsychotic-induced weight gain
Source: NPJ Schizophr. 2019 Apr 10;5:7. doi: 10.1038/s41537-019-0075-y (PMC6458173; doi:10.1038/s41537-019-0075-y)
Supplement: Supplementary file 1 — Supplementary Tables S1-S4. [file 41537_2019_75_MOESM1_ESM.pdf]

Supplementary Table S1. Differential expression genes between the weight and the no weight gain groups before medication

geneID	Gene Symbol	Base Mean	Base Mean Weight Gain	Base Mean No Weight Gain	Fold Change	Log2 Fold Change	Pval	Padj	GeneRif Annotation
3047	HBG1	733,23	184,32	1282,13	6,96	2,80	6,25E-88	1,22E-83	CHOLEST
9381	OTOF	134,47	213,17	55,78	0,26	-1,93	7,64E-66	7,46E-62	
10964	IFI44L	2693,83	3789,40	1598,25	0,42	-1,25	6,04E-32	3,93E-28	
6614	SIGLEC1	1492,89	2042,72	943,07	0,46	-1,12	1,34E-29	6,56E-26	
91543	RSAD2	1816,43	2611,27	1021,60	0,39	-1,35	7,31E-29	2,85E-25	
54094	C21orf15	282,17	399,10	165,24	0,41	-1,27	2,09E-26	6,80E-23	
3048	HBG2	2444,10	1364,10	3524,10	2,58	1,37	5,53E-25	1,54E-21	CHOLEST
10529	NEBL	182,79	93,49	272,09	2,91	1,54	9,49E-25	2,32E-21	
10232	MSLN	72,54	112,72	32,35	0,29	-1,80	5,84E-22	1,27E-18	CHOLEST
6557	SLC12A1	2998,20	1888,86	4107,54	2,17	1,12	1,53E-20	2,98E-17	
3127	HLA-DRB5	5034,64	3668,96	6400,31	1,74	0,80	1,02E-18	1,82E-15	
57126	CD177	247,35	311,20	183,51	0,59	-0,76	9,37E-18	1,52E-14	
93979	CPA5	42,86	67,42	18,31	0,27	-1,88	2,26E-17	3,40E-14	
101927586	RP11-290F20.2	914,10	1177,29	650,90	0,55	-0,85	8,28E-17	1,15E-13	
389396	GLYATL3	4,99	0,00	9,97	#¡DIV/0!	#¡DIV/0!	1,01E-15	1,32E-12	
10561	IFI44	3833,70	4877,80	2789,61	0,57	-0,81	5,07E-15	6,13E-12	
23532	PRAME	13,87	25,82	1,92	0,07	-3,75	5,33E-15	6,13E-12	
3119	HLA-DQB1	5801,02	4576,46	7025,57	1,54	0,62	9,98E-15	1,08E-11	CHOLEST
55384	MEG3	79,95	46,75	113,16	2,42	1,28	2,15E-14	2,21E-11	
26807	SNORD43	420,61	510,22	330,99	0,65	-0,62	2,31E-14	2,25E-11	
284751	RP11-290F20.1	1943,24	2432,13	1454,36	0,60	-0,74	6,46E-14	6,00E-11	
284581	LOC284581	171,63	218,55	124,71	0,57	-0,81	7,46E-14	6,62E-11	
56603	CYP26B1	63,98	35,37	92,58	2,62	1,39	8,51E-14	7,23E-11	
64478	CSMD1	53,51	72,19	34,83	0,48	-1,05	1,10E-13	8,93E-11	
23026	MYO16	486,89	690,49	283,29	0,41	-1,29	3,79E-13	2,96E-10	
554226	ANKRD30BL	415,21	524,99	305,44	0,58	-0,78	4,00E-13	3,00E-10	
3488	IGFBP5	11,02	3,21	18,83	5,86	2,55	4,20E-12	3,04E-09	
3434	IFIT1	3027,77	3830,21	2225,33	0,58	-0,78	4,58E-12	3,20E-09	
91977	MYOZ3	12,10	19,54	4,65	0,24	-2,07	1,38E-11	9,32E-09	
5139	PDE3A	1204,61	1473,36	935,87	0,64	-0,65	2,17E-11	1,41E-08	
100423062	IGLL5	3580,81	2655,82	4505,81	1,70	0,76	4,18E-11	2,63E-08	
26011	TENM4	65,87	48,28	83,47	1,73	0,79	5,05E-11	3,08E-08	
1290	COL5A2	42,41	56,95	27,87	0,49	-1,03	6,67E-11	3,95E-08	
399697	CTXN2	64,58	40,26	88,89	2,21	1,14	9,95E-11	5,71E-08	
342184	FMN1	775,96	609,88	942,04	1,54	0,63	1,60E-10	8,91E-08	
710	SERPING1	781,93	979,17	584,68	0,60	-0,74	1,85E-10	1,01E-07	
131540	ZDHHC19	174,07	218,64	129,51	0,59	-0,76	2,63E-10	1,39E-07	
116835	HSPA12B	24,42	13,21	35,62	2,70	1,43	3,35E-10	1,72E-07	
7225	TRPC6	68,44	90,66	46,22	0,51	-0,97	3,87E-10	1,94E-07	
1690	COCH	267,16	201,85	332,46	1,65	0,72	3,99E-10	1,95E-07	
129607	CMPK2	1017,72	1261,31	774,14	0,61	-0,70	5,29E-10	2,52E-07	
3429	IFI27	139,66	140,28	139,04	0,99	-0,01	1,11E-09	5,18E-07	
100506071	RP11-829H16.3	213,21	160,66	265,76	1,65	0,73	1,85E-09	8,39E-07	
3437	IFIT3	6360,64	7830,66	4890,62	0,62	-0,68	2,55E-09	1,13E-06	
54097	FAM3B	49,56	60,95	38,17	0,63	-0,68	3,14E-09	1,36E-06	
10398	MYL9	834,15	990,53	677,78	0,68	-0,55	5,02E-09	2,13E-06	
91181	NUP210L	51,66	72,93	30,39	0,42	-1,26	6,19E-09	2,52E-06	
283726	FAM154B	102,44	59,07	145,82	2,47	1,30	6,20E-09	2,52E-06	
51673	TPPP3	304,77	247,10	362,44	1,47	0,55	7,15E-09	2,85E-06	
1832	DSP	191,28	143,26	239,29	1,67	0,74	9,91E-09	3,87E-06	
346171	ZFP57	67,38	46,75	88,01	1,88	0,91	1,02E-08	3,91E-06	
266727	MDGA1	564,69	621,92	507,46	0,82	-0,29	1,17E-08	4,38E-06	
165530	CLEC4F	143,83	99,17	188,48	1,90	0,93	1,35E-08	4,99E-06	
728577	CNTNAP3B	29,89	13,99	45,80	3,27	1,71	1,53E-08	5,52E-06	
608	TNFRSF17	204,68	152,59	256,76	1,68	0,75	1,62E-08	5,74E-06	
101059918	GOLGA8R	54,00	71,85	36,14	0,50	-0,99	1,92E-08	6,70E-06	
7849	PAX8	1597,48	1319,63	1875,33	1,42	0,51	2,50E-08	8,58E-06	

338785	KRT79	12,19	2,96	21,42	7,24	2,86	2,60E-08	8,74E-06	
654433	PAX8-AS1	1299,21	1074,52	1523,91	1,42	0,50	3,08E-08	1,02E-05	
116071	BATF2	282,90	356,59	209,22	0,59	-0,77	4,32E-08	1,41E-05	
3486	IGFBP3	219,99	166,21	273,76	1,65	0,72	6,17E-08	1,97E-05	BMI, CHOLEST
94240	EPSTI1	2150,60	2570,83	1730,37	0,67	-0,57	9,18E-08	2,89E-05	
219970	GLYATL2	10,27	2,69	17,85	6,63	2,73	1,06E-07	3,28E-05	
5266	PI3	1026,76	1259,64	793,89	0,63	-0,67	1,11E-07	3,40E-05	
266629	SEC14L3	61,33	78,37	44,29	0,57	-0,82	1,87E-07	5,62E-05	
4747	NEFL	201,83	160,58	243,08	1,51	0,60	1,91E-07	5,66E-05	
5473	PPBP	3712,08	4424,74	2999,43	0,68	-0,56	2,02E-07	5,88E-05	
55966	AJAP1	208,82	236,02	181,62	0,77	-0,38	2,84E-07	8,15E-05	
29126	CD274	709,53	861,00	558,06	0,65	-0,63	3,14E-07	8,88E-05	
100272216	LOC100272216	544,37	663,58	425,16	0,64	-0,64	3,22E-07	8,99E-05	
1396	CRIP1	3572,88	2954,29	4191,47	1,42	0,50	3,34E-07	9,19E-05	
3904	LAIR2	78,01	61,85	94,17	1,52	0,61	3,56E-07	9,65E-05	
338773	TMEM119	70,46	91,69	49,22	0,54	-0,90	4,04E-07	1,08E-04	
6007	RHD	1045,15	1237,18	853,12	0,69	-0,54	4,39E-07	1,16E-04	
7130	TNFAIP6	910,64	1084,30	736,98	0,68	-0,56	5,32E-07	1,39E-04	
144195	SLC2A14	105,63	132,16	79,10	0,60	-0,74	6,20E-07	1,59E-04	
642846	LOC642846	307,36	361,81	252,91	0,70	-0,52	8,09E-07	2,05E-04	
9828	ARHGEF17	120,45	152,42	88,47	0,58	-0,78	8,27E-07	2,07E-04	
101927780	RP11-47I22.2	143,24	169,17	117,32	0,69	-0,53	1,06E-06	2,61E-04	
118932	ANKRD22	242,76	295,17	190,34	0,64	-0,63	1,13E-06	2,74E-04	
114990	VASN	125,37	155,27	95,47	0,61	-0,70	1,14E-06	2,74E-04	
3911	LAMA5	282,62	230,02	335,22	1,46	0,54	1,15E-06	2,75E-04	
114132	SIGLEC11	78,70	105,27	52,14	0,50	-1,01	1,19E-06	2,81E-04	
27181	SIGLEC8	434,03	461,02	407,05	0,88	-0,18	1,26E-06	2,94E-04	
55007	FAM118A	2253,89	2666,98	1840,81	0,69	-0,53	1,31E-06	3,01E-04	
5627	PROS1	102,16	127,72	76,60	0,60	-0,74	1,34E-06	3,04E-04	
3050	HBZ	39,57	26,33	52,81	2,01	1,00	1,61E-06	3,60E-04	
1397	CRIP2	491,22	392,87	589,57	1,50	0,59	2,10E-06	4,65E-04	
10410	IFITM3	8811,05	9903,60	7718,50	0,78	-0,36	2,46E-06	5,39E-04	
23495	TNFRSF13B	201,96	162,86	241,05	1,48	0,57	2,79E-06	6,04E-04	
3690	ITGB3	2175,58	2618,18	1732,98	0,66	-0,60	2,88E-06	6,17E-04	CHOLEST
114884	OSBPL10	546,00	449,03	642,98	1,43	0,52	3,06E-06	6,47E-04	CHOLEST
25849	PARM1	221,41	177,37	265,44	1,50	0,58	3,11E-06	6,47E-04	
64105	CENPK	814,50	938,98	690,01	0,73	-0,44	3,10E-06	6,47E-04	
9945	GFPT2	33,74	22,57	44,92	1,99	0,99	3,29E-06	6,76E-04	
3512	IGJ	5769,70	4746,53	6792,88	1,43	0,52	3,38E-06	6,88E-04	
81567	TXNDC5	6644,15	5474,20	7814,10	1,43	0,51	4,11E-06	8,27E-04	
440387	CTRB2	14,78	17,55	12,01	0,68	-0,55	4,72E-06	9,40E-04	
100506159	LOC100506159	141,00	166,70	115,30	0,69	-0,53	4,80E-06	9,46E-04	
219736	STOX1	38,31	51,32	25,30	0,49	-1,02	4,88E-06	9,53E-04	
838	CASP5	635,12	738,38	531,85	0,72	-0,47	5,46E-06	1,06E-03	
1824	DSC2	1676,05	1975,30	1376,81	0,70	-0,52	5,81E-06	1,11E-03	
283358	B4GALNT3	225,17	183,62	266,73	1,45	0,54	6,16E-06	1,17E-03	
654341	TBC1D3G	3,27	0,22	6,31	28,39	4,83	6,40E-06	1,20E-03	
54739	XAF1	6462,84	7370,73	5554,96	0,75	-0,41	6,64E-06	1,23E-03	
4061	LY6E	5351,50	6055,24	4647,76	0,77	-0,38	6,98E-06	1,29E-03	
1286	COL4A4	201,37	162,32	240,42	1,48	0,57	7,54E-06	1,38E-03	
66000	TMEM108	138,32	171,77	104,87	0,61	-0,71	8,35E-06	1,51E-03	
2487	FRZB	3,42	0,59	6,26	10,62	3,41	8,57E-06	1,53E-03	
64284	RAB17	8,32	3,11	13,53	4,35	2,12	1,09E-05	1,94E-03	
360226	PRSS41	93,49	103,71	83,27	0,80	-0,32	1,26E-05	2,21E-03	
79365	BHLHE41	157,11	126,13	188,08	1,49	0,58	1,33E-05	2,30E-03	
23428	SLC7A8	188,98	225,33	152,63	0,68	-0,56	1,33E-05	2,30E-03	
2258	FGF13	82,65	101,12	64,19	0,63	-0,66	1,37E-05	2,35E-03	
10501	SEMA6B	95,69	113,95	77,42	0,68	-0,56	1,40E-05	2,37E-03	
140	ADORA3	495,95	555,36	436,55	0,79	-0,35	1,43E-05	2,40E-03	
8082	SSPN	204,12	168,22	240,01	1,43	0,51	1,67E-05	2,78E-03	
3816	KLK1	36,96	27,43	46,48	1,69	0,76	1,68E-05	2,78E-03	
3620	IDO1	288,37	324,00	252,74	0,78	-0,36	1,81E-05	2,97E-03	BMI
10178	TENM1	772,78	887,77	657,79	0,74	-0,43	1,89E-05	3,07E-03	

116369	SLC26A8	907,17	1062,32	752,01	0,71	-0,50	1,93E-05	3,11E-03	
440068	CARD17	181,48	212,22	150,73	0,71	-0,49	2,05E-05	3,28E-03	
200958	MUC20	167,82	186,57	149,06	0,80	-0,32	2,11E-05	3,34E-03	
84680	ACCS	1326,71	1479,76	1173,65	0,79	-0,33	2,37E-05	3,73E-03	
30848	CTAG2	2,29	0,33	4,25	12,72	3,67	2,68E-05	4,18E-03	
4940	OAS3	5566,88	6467,27	4666,49	0,72	-0,47	2,79E-05	4,33E-03	
222389	BEND7	139,56	172,23	106,89	0,62	-0,69	2,83E-05	4,35E-03	
57699	CPNE5	1123,07	942,26	1303,87	1,38	0,47	2,92E-05	4,46E-03	
2791	GNG11	997,89	1148,51	847,26	0,74	-0,44	3,17E-05	4,79E-03	
5891	MOK	77,97	64,48	91,47	1,42	0,50	3,81E-05	5,72E-03	
3934	LCN2	807,57	625,96	989,19	1,58	0,66	3,84E-05	5,73E-03	BMI
2209	FCGR1A	857,96	996,45	719,48	0,72	-0,47	3,88E-05	5,74E-03	
23639	LRRC6	884,40	816,99	951,80	1,17	0,22	3,97E-05	5,82E-03	
10562	OLFM4	306,85	240,59	373,11	1,55	0,63	4,12E-05	6,00E-03	
145645	C15orf43	53,37	64,01	42,72	0,67	-0,58	4,18E-05	6,05E-03	
3674	ITGA2B	2241,36	2582,62	1900,10	0,74	-0,44	4,22E-05	6,06E-03	
9242	MSC	137,17	112,76	161,57	1,43	0,52	4,40E-05	6,26E-03	
10215	OLIG2	138,63	149,26	128,00	0,86	-0,22	4,48E-05	6,33E-03	
973	CD79A	3978,03	3399,70	4556,35	1,34	0,42	4,63E-05	6,51E-03	
714	C1QC	38,73	45,18	32,28	0,71	-0,49	4,80E-05	6,64E-03	
2838	GPR15	577,94	543,24	612,63	1,13	0,17	4,77E-05	6,64E-03	
3123	HLA-DRB1	10360,94	9235,92	11485,96	1,24	0,31	4,97E-05	6,83E-03	CHOLEST
9891	NUAK1	38,56	26,26	50,86	1,94	0,95	5,06E-05	6,91E-03	
3092	HIP1	4048,84	4490,19	3607,49	0,80	-0,32	5,14E-05	6,97E-03	
2537	IFI6	2101,11	2393,51	1808,70	0,76	-0,40	5,63E-05	7,56E-03	
100526836	BLOC1S5-TXNDC5	7661,76	6488,70	8834,83	1,36	0,45	5,65E-05	7,56E-03	
84623	KIRREL3	207,59	234,16	181,03	0,77	-0,37	5,75E-05	7,64E-03	
168620	BHLHA15	19,90	13,42	26,38	1,97	0,98	5,81E-05	7,66E-03	
105	ADARB2	957,15	1047,66	866,63	0,83	-0,27	6,02E-05	7,88E-03	
728262	FAM157A	745,88	849,11	642,64	0,76	-0,40	6,05E-05	7,88E-03	
10395	DLC1	39,26	26,41	52,11	1,97	0,98	6,12E-05	7,92E-03	
11346	SYNPO	344,11	292,50	395,72	1,35	0,44	6,21E-05	7,97E-03	
4605	MYBL2	221,22	175,33	267,11	1,52	0,61	6,26E-05	7,99E-03	
3938	LCT	25,89	31,86	19,93	0,63	-0,68	7,15E-05	9,06E-03	BMI
51237	MZB1	814,75	676,88	952,61	1,41	0,49	7,46E-05	9,40E-03	

Headers of the Table	
geneID	Gene Identification
Gene Symbol	Official Symbol
Base Mean	Mean normalized counts, averaged over all samples from both conditions
Base Mean Weight Gain	Mean normalized counts from condition A
Base Mean No Weight Gain	Mean normalized counts from condition B
Fold Change	Fold change from condition A to B (B/A)
Log2 Fold Change	The logarithm, to basis 2, of the fold change
Pval	P value for the statistical significance of this change
Padj	P value adjusted for multiple testing with the Benjamini-Hochberg procedure, which controls false discovery rate
GeneRif Annotation	Genes including the strings “BMI” or “cholest” in their GeneRIF definition

Supplementary Table S2. Differential expression genes before and after 3 months of antipsychotic medication in the weight gain group

geneID	Gene Symbol	Base Mean	Base Mean Before Medication	Base Mean After Medication	Fold Change	Log2 Fold Change	Pval	Padj	GeneRif Annotation
100462981	MTRNR2L2	259.65	24.81	494.49	19.93	4.32	1.68E-139	3.27E-135	
3429	IFI27	75.87	127.06	24.68	0.19	-2.36	1.61E-55	1.56E-51	
9381	OTOF	126.75	193.19	60.32	0.31	-1.68	3.62E-49	2.34E-45	
100190986	LOC100190986	49.18	8.81	89.54	10.17	3.35	1.74E-41	8.45E-38	
10562	OLFM4	411.37	217.58	605.17	2.78	1.48	1.94E-37	7.53E-34	
9509	ADAMTS2	43.10	72.83	13.38	0.18	-2.44	1.14E-36	3.68E-33	
4057	LTF	3526.72	2039.45	5013.98	2.46	1.30	5.59E-35	1.55E-31	
3934	LCN2	974.95	566.25	1383.66	2.44	1.29	4.24E-34	1.03E-30	BMI
4317	MMP8	496.57	297.24	695.90	2.34	1.23	3.15E-30	6.80E-27	CHOLEST
154664	ABCA13	277.61	155.82	399.40	2.56	1.36	5.00E-30	9.71E-27	
1088	CEACAM8	556.83	321.14	792.53	2.47	1.30	1.96E-29	3.47E-26	
10321	CRISP3	265.27	163.34	367.21	2.25	1.17	7.19E-24	1.16E-20	
4973	OLR1	84.71	46.21	123.20	2.67	1.41	5.58E-22	8.34E-19	CHOLEST
1667	DEFA1	2098.89	1385.07	2812.72	2.03	1.02	5.33E-21	6.47E-18	CHOLEST
728358	DEFA1B	2098.89	1385.07	2812.72	2.03	1.02	5.33E-21	6.47E-18	
1668	DEFA3	2098.89	1385.07	2812.72	2.03	1.02	5.33E-21	6.47E-18	CHOLEST
4680	CEACAM6	422.66	267.73	577.58	2.16	1.11	2.54E-20	2.90E-17	
23500	DAAM2	428.62	586.27	270.96	0.46	-1.11	6.56E-20	7.08E-17	
1669	DEFA4	443.69	285.76	601.61	2.11	1.07	3.12E-19	3.19E-16	
6614	SIGLEC1	1438.49	1849.10	1027.87	0.56	-0.85	2.13E-17	2.07E-14	
671	BPI	983.89	689.83	1277.95	1.85	0.89	1.85E-15	1.71E-12	
10964	IFI44L	2680.65	3429.55	1931.74	0.56	-0.83	3.25E-15	2.87E-12	
6948	TCN2	326.53	415.37	237.69	0.57	-0.81	7.00E-13	5.92E-10	
4353	MPO	930.24	667.37	1193.10	1.79	0.84	8.63E-13	6.99E-10	BMI, CHOLEST
246	ALOX15	1331.00	966.12	1695.88	1.76	0.81	7.69E-12	5.98E-09	CHOLEST
5139	PDE3A	1099.33	1333.58	865.08	0.65	-0.62	1.95E-11	1.46E-08	
1511	CTSG	144.84	99.64	190.05	1.91	0.93	2.43E-11	1.75E-08	
932	MS4A3	558.95	424.58	693.33	1.63	0.71	2.38E-10	1.65E-07	
5657	PRTN3	82.14	53.77	110.51	2.06	1.04	2.69E-10	1.81E-07	
260429	PRSS33	716.35	538.32	894.38	1.66	0.73	4.73E-10	3.07E-07	
26807	SNORD43	440.45	462.31	418.59	0.91	-0.14	5.80E-10	3.64E-07	
27181	SIGLEC8	551.89	417.11	686.67	1.65	0.72	9.22E-10	5.60E-07	
554226	ANKRD30BL	392.51	475.10	309.92	0.65	-0.62	2.14E-09	1.26E-06	
713	C1QB	73.59	92.98	54.20	0.58	-0.78	3.22E-09	1.84E-06	
2078	ERG	76.32	52.20	100.44	1.92	0.94	4.98E-09	2.77E-06	BMI, CHOLEST
820	CAMP	658.36	519.29	797.44	1.54	0.62	5.59E-09	3.02E-06	
5819	PVRL2	348.80	395.56	302.04	0.76	-0.39	7.54E-09	3.96E-06	CHOLEST
1991	ELANE	257.88	188.35	327.40	1.74	0.80	9.32E-09	4.77E-06	
10410	IFITM3	7407.41	8964.88	5849.94	0.65	-0.62	1.22E-08	6.08E-06	
387755	INSC	93.23	120.81	65.65	0.54	-0.88	2.01E-08	9.53E-06	
212	ALAS2	5696.89	7086.14	4307.64	0.61	-0.72	1.98E-08	9.53E-06	
1308	COL17A1	57.85	39.87	75.82	1.90	0.93	4.84E-08	2.24E-05	
55301	OLAH	27.90	39.08	16.71	0.43	-1.23	5.38E-08	2.43E-05	
19	ABCA1	3549.63	4191.95	2907.32	0.69	-0.53	6.32E-08	2.79E-05	BMI, CHOLEST
3620	IDO1	372.40	293.14	451.67	1.54	0.62	6.55E-08	2.83E-05	BMI
566	AZU1	323.03	244.01	402.06	1.65	0.72	7.99E-08	3.33E-05	
91543	RSAD2	1904.06	2362.04	1446.08	0.61	-0.71	8.06E-08	3.33E-05	
9672	SDC3	236.36	288.74	183.99	0.64	-0.65	9.66E-08	3.91E-05	
94031	HTRA3	22.87	14.14	31.60	2.23	1.16	2.52E-07	0.00010	
6947	TCN1	273.00	218.68	327.32	1.50	0.58	4.95E-07	0.00019	CHOLEST
2030	SLC29A1	1128.86	910.51	1347.21	1.48	0.57	5.27E-07	0.00020	
4481	MSR1	241.92	288.65	195.18	0.68	-0.56	5.22E-07	0.00020	
283120	H19	27.11	17.35	36.87	2.13	1.09	5.37E-07	0.00020	BMI, CHOLEST
100133941	CD24	1378.05	1132.02	1624.08	1.43	0.52	6.03E-07	0.00022	
7130	TNFAIP6	829.02	981.59	676.45	0.69	-0.54	6.76E-07	0.00024	
2359	FPR3	216.02	263.34	168.70	0.64	-0.64	7.45E-07	0.00026	
83869	TTTY14	1402.58	1154.14	1651.01	1.43	0.52	7.67E-07	0.00026	
714	C1QC	32.25	40.90	23.61	0.58	-0.79	7.97E-07	0.00027	

6037	RNASE3	226.54	172.08	281.01	1.63	0.71	9.91E-07	0.00033	BMI
2168	FABP1	1.87	3.64	0.10	0.03	-5.22	1.17E-06	0.00037	CHOLEST
5166	PDK4	1536.58	1800.70	1272.45	0.71	-0.50	1.16E-06	0.00037	
712	C1QA	132.57	156.71	108.43	0.69	-0.53	1.25E-06	0.00039	
26577	PCOLCE2	12.66	6.48	18.83	2.90	1.54	1.27E-06	0.00039	CHOLEST
10215	OLIG2	173.74	135.04	212.44	1.57	0.65	1.40E-06	0.00042	
1053	CEBPE	340.64	272.41	408.87	1.50	0.59	1.92E-06	0.00057	
762	CA4	626.78	737.77	515.80	0.70	-0.52	1.99E-06	0.00059	
79056	PRRG4	1358.84	1591.43	1126.26	0.71	-0.50	2.12E-06	0.00062	
23026	MYO16	506.08	625.44	386.71	0.62	-0.69	2.19E-06	0.00063	
54682	MANSC1	1778.93	2062.89	1494.98	0.72	-0.46	2.51E-06	0.00071	
1178	CLC	4661.99	3848.79	5475.19	1.42	0.51	3.16E-06	0.00088	
11026	LILRA3	1935.75	2239.16	1632.35	0.73	-0.46	3.42E-06	0.00094	
6035	RNASE1	30.88	41.11	20.65	0.50	-0.99	4.06E-06	0.00110	
4583	MUC2	1.74	3.39	0.09	0.03	-5.24	4.58E-06	0.00121	
133	ADM	1189.76	1385.46	994.05	0.72	-0.48	4.66E-06	0.00121	BMI
144195	SLC2A14	99.52	119.71	79.33	0.66	-0.59	4.62E-06	0.00121	
10720	UGT2B11	642.39	526.64	758.14	1.44	0.53	4.82E-06	0.00123	
57126	CD177	241.33	281.67	200.99	0.71	-0.49	5.17E-06	0.00127	
101927586	RP11-290F20.2	942.66	1065.73	819.60	0.77	-0.38	5.10E-06	0.00127	
8460	TPST1	631.02	749.84	512.19	0.68	-0.55	5.14E-06	0.00127	
22895	RPH3A	481.41	534.53	428.29	0.80	-0.32	5.75E-06	0.00140	
1179	CLCA1	1.66	3.21	0.12	0.04	-4.78	7.77E-06	0.00187	
199675	C19orf59	829.20	957.20	701.19	0.73	-0.45	8.32E-06	0.00197	
6768	ST14	1102.79	1263.81	941.76	0.75	-0.42	8.63E-06	0.00200	
94	ACVRL1	65.20	80.52	49.88	0.62	-0.69	8.65E-06	0.00200	
1719	DHFR	913.24	674.56	1151.92	1.71	0.77	9.46E-06	0.00216	
10461	MERTK	307.03	359.17	254.89	0.71	-0.49	1.04E-05	0.00234	
2322	FLT3	574.20	664.73	483.67	0.73	-0.46	1.34E-05	0.00299	
653061	GOLGA8S	64.35	42.69	86.01	2.01	1.01	1.42E-05	0.00313	
79937	CNTNAP3	1609.66	1868.71	1350.60	0.72	-0.47	1.43E-05	0.00313	
4061	LY6E	4802.64	5480.03	4125.25	0.75	-0.41	1.62E-05	0.00349	
83999	KREMEN1	1996.29	2310.36	1682.21	0.73	-0.46	1.85E-05	0.00396	
722	C4BPA	101.42	80.22	122.62	1.53	0.61	2.00E-05	0.00423	
440068	CARD17	164.15	192.13	136.17	0.71	-0.50	2.10E-05	0.00440	
717	C2	261.31	306.48	216.14	0.71	-0.50	2.14E-05	0.00442	
8942	KYNU	828.94	964.88	693.01	0.72	-0.48	2.20E-05	0.00451	
9447	AIM2	830.82	961.13	700.51	0.73	-0.46	2.29E-05	0.00461	
5730	PTGDS	759.11	837.56	680.67	0.81	-0.30	2.30E-05	0.00461	
731424	RP11-701P16.5	351.48	401.08	301.88	0.75	-0.41	2.65E-05	0.00526	
2687	GGT5	71.69	55.76	87.62	1.57	0.65	2.93E-05	0.00570	
10917	BTNL3	483.21	548.85	417.58	0.76	-0.39	2.93E-05	0.00570	
100463486	MTRNR2L8	115.27	82.31	148.23	1.80	0.85	2.99E-05	0.00576	
765	CA6	823.20	717.39	929.00	1.29	0.37	3.19E-05	0.00608	
353514	LILRA5	3611.27	4161.00	3061.54	0.74	-0.44	3.26E-05	0.00615	
10501	SEMA6B	87.20	103.14	71.25	0.69	-0.53	3.71E-05	0.00693	
6280	S100A9	133237.54	151778.95	114696.12	0.76	-0.40	3.79E-05	0.00701	
51338	MS4A4A	220.38	260.41	180.35	0.69	-0.53	4.21E-05	0.00772	
284751	RP11-290F20.1	1978.17	2201.62	1754.72	0.80	-0.33	4.36E-05	0.00793	
10561	IFI44	3836.21	4414.41	3258.02	0.74	-0.44	4.53E-05	0.00816	
1118	CHIT1	130.26	104.29	156.23	1.50	0.58	4.78E-05	0.00847	
10184	LHFPL2	903.15	1045.05	761.25	0.73	-0.46	4.81E-05	0.00847	
100874264	AOAH-IT1	31.86	42.10	21.61	0.51	-0.96	4.88E-05	0.00847	
8972	MGAM	14037.22	15917.41	12157.04	0.76	-0.39	4.85E-05	0.00847	
11326	VSIG4	160.92	190.45	131.38	0.69	-0.54	5.12E-05	0.00880	
55512	SMPD3	1959.32	1660.44	2258.20	1.36	0.44	5.18E-05	0.00883	
11251	PTGDR2	1025.69	876.52	1174.85	1.34	0.42	5.77E-05	0.00976	

Headers of the Table	
geneID	Gene Identification
Gene Symbol	Official Symbol
Base Mean	Mean normalized counts, averaged over all samples from both conditions

Base Mean Before Medication	Mean normalized counts from condition A
Base Mean After Medication	Mean normalized counts from condition B
Fold Change	Fold change from condition A to B (B/A)
Log2 Fold Change	The logarithm, to basis 2, of the fold change
Pval	P value for the statistical significance of this change
Padj	P value adjusted for multiple testing with the Benjamini-Hochberg procedure, which controls false discovery rate
GeneRif Annotation	Genes including the strings “BMI” or “cholest” in their GeneRIF definition

Supplementary Table S3. Differential expression genes before and after 3 months of antipsychotic medication in the no weight gain group

geneID	Gene Symbol	Base Mean	Base Mean Before Medication	Base Mean After Medication	Fold Change	Log2 Fold Change	Pval	Padj	GeneRif Annotation
100462981	MTRNR2L2	26,80	1108,97	41,38	41,38	5,37	2,34E-226	4,57E-222	
100190986	LOC100190986	9,60	112,72	11,74	11,74	3,55	3,12E-52	3,05E-48	
3620	IDO1	248,04	592,70	2,39	2,39	1,26	1,19E-39	7,77E-36	BMI
118932	ANKRD22	186,84	437,44	2,34	2,34	1,23	1,62E-31	7,92E-28	
9509	ADAMTS2	84,65	20,24	0,24	0,24	-2,06	6,95E-31	2,71E-27	
3627	CXCL10	82,27	208,90	2,54	2,54	1,34	7,59E-30	2,47E-26	
419	ART3	90,41	223,32	2,47	2,47	1,30	2,01E-29	5,61E-26	
1719	DHFR	704,83	1808,97	2,57	2,57	1,36	1,87E-25	4,56E-22	
115362	GBP5	10500,80	19955,17	1,90	1,90	0,93	1,19E-24	2,58E-21	
710	SERPING1	573,69	1107,86	1,93	1,93	0,95	2,83E-24	5,52E-21	
116071	BATF2	205,30	426,03	2,08	2,08	1,05	5,56E-23	9,88E-20	
51513	ETV7	186,93	412,00	2,20	2,20	1,14	6,72E-23	1,09E-19	
100463486	MTRNR2L8	86,68	251,67	2,90	2,90	1,54	5,13E-22	7,70E-19	
91543	RSAD2	1002,32	1776,90	1,77	1,77	0,83	2,89E-21	4,03E-18	
246	ALOX15	1283,03	1848,49	1,44	1,44	0,53	8,26E-17	1,08E-13	CHOLEST
80832	APOL4	60,12	129,39	2,15	2,15	1,11	9,73E-17	1,19E-13	
100423062	IGLL5	4421,30	2416,72	0,55	0,55	-0,87	1,04E-16	1,19E-13	
10501	SEMA6B	76,07	167,70	2,20	2,20	1,14	3,40E-16	3,69E-13	
27181	SIGLEC8	399,48	601,42	1,51	1,51	0,59	3,74E-16	3,84E-13	
9934	P2RY14	649,86	1051,01	1,62	1,62	0,69	4,42E-16	4,31E-13	
23500	DAAM2	569,82	393,31	0,69	0,69	-0,53	1,77E-15	1,65E-12	
1278	COL1A2	4,52	20,33	4,49	4,49	2,17	3,70E-15	3,28E-12	
2633	GBP1	4104,47	6675,87	1,63	1,63	0,70	7,00E-15	5,94E-12	
27074	LAMP3	133,19	237,12	1,78	1,78	0,83	7,85E-15	6,39E-12	
79937	CNTNAP3	2160,13	1486,05	0,69	0,69	-0,54	1,36E-14	1,06E-11	
10964	IFI44L	1567,98	2457,47	1,57	1,57	0,65	2,57E-14	1,93E-11	
3429	IFI27	136,60	48,26	0,35	0,35	-1,50	2,27E-13	1,64E-10	
54094	C21orf15	162,16	268,61	1,66	1,66	0,73	7,78E-13	5,42E-10	
260429	PRSS33	582,28	783,97	1,35	1,35	0,43	1,30E-12	8,74E-10	
115361	GBP4	4483,67	6817,04	1,52	1,52	0,60	5,33E-12	3,47E-09	
146439	CCDC64B	281,32	169,26	0,60	0,60	-0,73	3,50E-11	2,20E-08	
55512	SMPD3	1610,28	2239,14	1,39	1,39	0,48	5,83E-11	3,56E-08	
26807	SNORD43	324,73	150,11	0,46	0,46	-1,11	7,54E-11	4,46E-08	
4283	CXCL9	25,20	55,36	2,20	2,20	1,14	1,40E-10	8,04E-08	
3048	HBG2	3463,51	1928,19	0,56	0,56	-0,84	2,82E-10	1,57E-07	CHOLEST
3512	IGJ	6667,05	4285,95	0,64	0,64	-0,64	6,33E-10	3,43E-07	
29126	CD274	547,72	813,34	1,48	1,48	0,57	8,72E-10	4,60E-07	
10720	UGT2B11	522,54	747,06	1,43	1,43	0,52	9,86E-10	5,07E-07	
5990	RFX2	1532,49	1109,99	0,72	0,72	-0,47	1,10E-09	5,49E-07	
387755	INSC	121,87	68,55	0,56	0,56	-0,83	1,44E-09	6,85E-07	

3568	IL5RA	713,14	951,92	1,33	1,33	0,42	1,44E-09	6,85E-07	
608	TNFRSF17	252,05	144,79	0,57	0,57	-0,80	2,35E-09	1,09E-06	
7225	TRPC6	45,37	84,12	1,85	1,85	0,89	6,06E-09	2,75E-06	
59283	CACNG8	188,19	260,43	1,38	1,38	0,47	6,44E-09	2,86E-06	
27087	B3GAT1	591,48	874,00	1,48	1,48	0,56	9,23E-09	3,93E-06	
51673	TPPP3	355,79	247,39	0,70	0,70	-0,52	9,26E-09	3,93E-06	
3823	KLRC3	638,74	935,21	1,46	1,46	0,55	1,04E-08	4,33E-06	
467	ATF3	60,78	105,53	1,74	1,74	0,80	1,86E-08	7,55E-06	CHOLEST
51237	MZB1	934,69	603,45	0,65	0,65	-0,63	2,66E-08	1,06E-05	
10561	IFI44	2736,70	3780,68	1,38	1,38	0,47	2,77E-08	1,08E-05	
249	ALPL	10622,22	7638,33	0,72	0,72	-0,48	3,53E-08	1,35E-05	CHOLEST
81567	TXNDC5	7668,61	5140,36	0,67	0,67	-0,58	4,29E-08	1,61E-05	
1178	CLC	4252,57	5215,03	1,23	1,23	0,29	6,85E-08	2,52E-05	
10215	OLIG2	125,59	171,07	1,36	1,36	0,45	7,43E-08	2,68E-05	
340654	LIPM	9,33	25,64	2,75	2,75	1,46	1,15E-07	4,09E-05	
343413	FCRL6	1834,08	2511,29	1,37	1,37	0,45	1,49E-07	5,20E-05	
199675	C19orf59	916,79	634,16	0,69	0,69	-0,53	1,84E-07	6,29E-05	
51348	KLRF1	2398,33	3313,26	1,38	1,38	0,47	2,59E-07	8,70E-05	
8302	KLRC4	381,39	524,92	1,38	1,38	0,46	2,70E-07	8,92E-05	
2030	SLC29A1	911,04	1177,44	1,29	1,29	0,37	2,77E-07	9,00E-05	
5159	PDGFRB	310,72	426,35	1,37	1,37	0,46	3,24E-07	1,04E-04	
7453	WARS	9217,57	12071,43	1,31	1,31	0,39	3,60E-07	1,13E-04	
360226	PRSS41	81,69	112,62	1,38	1,38	0,46	3,99E-07	1,24E-04	
59340	HRH4	298,18	410,71	1,38	1,38	0,46	4,65E-07	1,42E-04	
129607	CMPK2	759,54	1013,61	1,33	1,33	0,42	5,08E-07	1,52E-04	
1281	COL3A1	3,36	10,72	3,19	3,19	1,68	5,50E-07	1,63E-04	CHOLEST
2735	GLI1	1364,30	1058,90	0,78	0,78	-0,37	6,76E-07	1,91E-04	
1277	COL1A1	9,17	20,72	2,26	2,26	1,18	6,76E-07	1,91E-04	
5414	SEPT4	85,21	138,05	1,62	1,62	0,70	6,69E-07	1,91E-04	
10417	SPON2	4800,57	6149,92	1,28	1,28	0,36	6,97E-07	1,94E-04	
169355	IDO2	32,11	55,53	1,73	1,73	0,79	1,17E-06	3,23E-04	
4318	MMP9	5448,93	3902,03	0,72	0,72	-0,48	1,26E-06	3,41E-04	BMI, CHOLEST
722	C4BPA	106,02	175,99	1,66	1,66	0,73	1,34E-06	3,59E-04	
140	ADORA3	428,57	550,41	1,28	1,28	0,36	1,39E-06	3,66E-04	
91663	MYADM	16747,27	13139,11	0,78	0,78	-0,35	1,40E-06	3,66E-04	
94240	EPSTI1	1697,82	2186,08	1,29	1,29	0,36	1,72E-06	4,43E-04	
54658	UGT1A1	4,11	0,11	0,03	0,03	-5,24	1,95E-06	4,93E-04	BMI
1634	DCN	2,52	10,17	4,04	4,04	2,01	2,08E-06	5,21E-04	
125875	CLDND2	275,26	387,46	1,41	1,41	0,49	2,16E-06	5,33E-04	
60675	PROK2	7658,56	5655,89	0,74	0,74	-0,44	2,19E-06	5,33E-04	
10316	NMUR1	897,47	1192,55	1,33	1,33	0,41	2,22E-06	5,34E-04	
100128770	RP11-473M20.5	501,30	366,43	0,73	0,73	-0,45	2,41E-06	5,73E-04	
100526836	BLOC1S5-TXNDC5	8670,39	6147,47	0,71	0,71	-0,50	2,44E-06	5,74E-04	
10409	BASP1	11301,95	8292,44	0,73	0,73	-0,45	2,58E-06	5,92E-04	
2172	FABP6	273,58	194,82	0,71	0,71	-0,49	2,55E-06	5,92E-04	
9050	PSTPIP2	3461,51	4383,66	1,27	1,27	0,34	2,71E-06	6,14E-04	
8671	SLC4A4	135,90	204,17	1,50	1,50	0,59	2,74E-06	6,14E-04	
100131561	FKSG29	58,43	81,27	1,39	1,39	0,48	2,92E-06	6,33E-04	
6772	STAT1	13774,49	18083,31	1,31	1,31	0,39	2,91E-06	6,33E-04	
54625	PARP14	14102,75	19031,89	1,35	1,35	0,43	2,91E-06	6,33E-04	
134549	SHROOM1	519,81	398,98	0,77	0,77	-0,38	3,04E-06	6,53E-04	
11251	PTGDR2	921,31	1129,48	1,23	1,23	0,29	3,10E-06	6,58E-04	
3816	KLK1	45,58	26,82	0,59	0,59	-0,77	3,22E-06	6,76E-04	
3488	IGFBP5	18,44	5,60	0,30	0,30	-1,72	4,65E-06	9,66E-04	
1844	DUSP2	765,67	1150,97	1,50	1,50	0,59	5,03E-06	1,03E-03	
653061	GOLGA8S	43,05	80,28	1,86	1,86	0,90	5,30E-06	1,08E-03	
283177	LOC283177	85,10	120,25	1,41	1,41	0,50	5,36E-06	1,08E-03	
338596	ST8SIA6	183,11	270,38	1,48	1,48	0,56	5,84E-06	1,15E-03	
4603	MYBL1	2843,69	3861,03	1,36	1,36	0,44	5,82E-06	1,15E-03	
9289	GPR56	6078,02	7843,57	1,29	1,29	0,37	6,12E-06	1,19E-03	
219537	SMTNL1	232,81	343,83	1,48	1,48	0,56	6,20E-06	1,20E-03	
4940	OAS3	4578,37	5898,12	1,29	1,29	0,37	7,55E-06	1,45E-03	
3824	KLRD1	2610,06	3505,34	1,34	1,34	0,43	7,82E-06	1,48E-03	

3802	KIR2DL1	184,43	251,33	1,36	1,36	0,45	9,38E-06	1,76E-03	
285696	AC091878.1	185,84	134,77	0,73	0,73	-0,46	9,79E-06	1,82E-03	
139189	DGKK	115,31	161,79	1,40	1,40	0,49	1,01E-05	1,87E-03	
3240	HP	273,03	178,99	0,66	0,66	-0,61	1,04E-05	1,90E-03	CHOLEST
3763	KCNJ6	786,34	611,49	0,78	0,78	-0,36	1,14E-05	2,06E-03	
54659	UGT1A3	4,17	0,19	0,05	0,05	-4,47	1,34E-05	2,40E-03	
1071	CETP	320,14	427,73	1,34	1,34	0,42	1,44E-05	2,56E-03	BMI, CHOLEST
125336	LOXHD1	460,90	366,39	0,79	0,79	-0,33	1,98E-05	3,48E-03	
80310	PDGFD	357,45	500,68	1,40	1,40	0,49	2,29E-05	3,95E-03	
1593	CYP27A1	2815,79	2182,03	0,77	0,77	-0,37	2,28E-05	3,95E-03	CHOLEST
54575	UGT1A10	6,61	0,99	0,15	0,15	-2,75	2,32E-05	3,98E-03	
54600	UGT1A9	6,59	0,99	0,15	0,15	-2,74	2,52E-05	4,27E-03	
54490	UGT2B28	38,05	55,61	1,46	1,46	0,55	2,54E-05	4,28E-03	
65061	CDK15	22,58	38,52	1,71	1,71	0,77	2,90E-05	4,84E-03	
101929452	AC019048.1	193,76	254,69	1,31	1,31	0,39	3,00E-05	4,96E-03	
59352	LGR6	518,12	658,59	1,27	1,27	0,35	3,13E-05	5,13E-03	
54576	UGT1A8	6,78	1,10	0,16	0,16	-2,62	3,25E-05	5,20E-03	
1232	CCR3	2642,45	3129,99	1,18	1,18	0,24	3,24E-05	5,20E-03	
441168	FAM26F	721,90	875,14	1,21	1,21	0,28	3,20E-05	5,20E-03	
5996	RGS1	119,71	181,85	1,52	1,52	0,60	3,32E-05	5,22E-03	
146857	SLFN13	1692,52	2153,12	1,27	1,27	0,35	3,34E-05	5,22E-03	
3809	KIR2DS4	235,84	319,89	1,36	1,36	0,44	3,33E-05	5,22E-03	
771	CA12	19,95	8,54	0,43	0,43	-1,22	3,63E-05	5,62E-03	
146433	IL34	43,34	61,06	1,41	1,41	0,49	3,74E-05	5,74E-03	
2999	GZMH	2585,30	3261,42	1,26	1,26	0,34	3,89E-05	5,93E-03	
2209	FCGR1A	706,23	890,36	1,26	1,26	0,33	4,00E-05	6,05E-03	
762	CA4	588,48	436,96	0,74	0,74	-0,43	4,16E-05	6,25E-03	
11326	VSIG4	224,12	167,31	0,75	0,75	-0,42	4,24E-05	6,32E-03	
55301	OLAH	35,13	17,81	0,51	0,51	-0,98	4,29E-05	6,35E-03	
401258	RAB44	962,70	1208,26	1,26	1,26	0,33	4,36E-05	6,39E-03	
57669	EPB41L5	365,79	471,82	1,29	1,29	0,37	4,46E-05	6,46E-03	
131540	ZDHHC19	127,09	176,31	1,39	1,39	0,47	4,50E-05	6,46E-03	
5166	PDK4	2026,38	1573,24	0,78	0,78	-0,37	4,47E-05	6,46E-03	
10578	GNLY	18321,50	22550,63	1,23	1,23	0,30	5,18E-05	7,32E-03	
1903	S1PR3	841,04	628,51	0,75	0,75	-0,42	5,15E-05	7,32E-03	
133396	IL31RA	75,42	116,30	1,54	1,54	0,62	5,29E-05	7,43E-03	
11098	PRSS23	683,00	878,96	1,29	1,29	0,36	5,47E-05	7,63E-03	
3913	LAMB2	266,87	201,98	0,76	0,76	-0,40	5,67E-05	7,85E-03	
3822	KLRC2	217,15	285,26	1,31	1,31	0,39	5,93E-05	8,10E-03	
54682	MANSC1	2036,09	1537,89	0,76	0,76	-0,40	5,90E-05	8,10E-03	
9173	IL1RL1	182,62	237,19	1,30	1,30	0,38	5,98E-05	8,11E-03	
9495	AKAP5	237,64	329,15	1,39	1,39	0,47	6,04E-05	8,13E-03	
54577	UGT1A7	6,22	0,99	0,16	0,16	-2,66	6,24E-05	8,29E-03	
54578	UGT1A6	6,22	0,99	0,16	0,16	-2,66	6,24E-05	8,29E-03	
2731	GLDC	84,20	52,62	0,62	0,62	-0,68	6,34E-05	8,37E-03	
56603	CYP26B1	90,90	69,50	0,76	0,76	-0,39	6,62E-05	8,67E-03	
266727	MDGA1	498,11	536,16	1,08	1,08	0,11	6,74E-05	8,73E-03	
9536	PTGES	37,01	20,84	0,56	0,56	-0,83	6,75E-05	8,73E-03	
84913	ATOH8	137,87	105,90	0,77	0,77	-0,38	7,19E-05	9,23E-03	
10562	OLFM4	365,64	448,03	1,23	1,23	0,29	7,51E-05	9,58E-03	
54739	XAF1	5450,26	6805,13	1,25	1,25	0,32	7,58E-05	9,60E-03	
80830	APOL6	8212,36	10468,38	1,27	1,27	0,35	7,91E-05	9,95E-03	
8277	TKTL1	303,31	353,60	1,17	1,17	0,22	7,95E-05	9,95E-03	

Headers of the Table	
geneID	Gene Identification
Gene Symbol	Official Symbol
Base Mean	Mean normalized counts, averaged over all samples from both conditions
Base Mean Before Medication	Mean normalized counts from condition A

Base Mean After Medication	Mean normalized counts from condition B
Fold Change	Fold change from condition A to B (B/A)
Log2 Fold Change	The logarithm, to basis 2, of the fold change
Pval	P value for the statistical significance of this change
Padj	P value adjusted for multiple testing with the Benjamini-Hochberg procedure, which controls false discovery rate
GeneRif Annotation	Genes including the strings “BMI” or “cholest” in their GeneRIF definition

Supplementary Table S4. Differential expression genes between the weight and the no weight gain groups after medication

geneID	Gene Symbol	Base Mean	Base Mean Weight Gain	Base Mean No Weight Gain	Fold Change	Log2 Fold Change	Pval	Padj	GeneRif Annotation
3047	HBG1	384,26	145,94	622,58	4,27	2,09	7,28E-78	1,42E-73	CHOLEST
100462981	MTRNR2L2	744,06	486,77	1001,34	2,06	1,04	3,81E-51	3,70E-47	
23532	PRAME	15,68	29,23	2,13	0,07	-3,78	1,99E-29	1,29E-25	
4317	MMP8	489,78	683,11	296,46	0,43	-1,20	1,04E-27	5,05E-24	CHOLEST
93979	CPA5	42,91	69,68	16,13	0,23	-2,11	1,47E-26	5,73E-23	
51513	ETV7	269,73	167,26	372,19	2,23	1,15	2,05E-26	6,64E-23	
118932	ANKRD22	293,26	191,43	395,08	2,06	1,05	2,34E-24	6,50E-21	
1278	COL1A2	9,76	1,18	18,34	15,60	3,96	5,33E-24	1,30E-20	
3127	HLA-DRB5	3798,38	2909,02	4687,75	1,61	0,69	1,28E-20	2,76E-17	
27087	B3GAT1	602,07	413,24	790,90	1,91	0,94	2,34E-20	4,55E-17	
26807	SNORD43	272,99	410,01	135,97	0,33	-1,59	3,15E-20	5,57E-17	
3848	KRT1	362,10	241,88	482,32	1,99	1,00	1,27E-19	2,06E-16	
10232	MSLN	60,01	83,02	37,01	0,45	-1,17	1,98E-19	2,96E-16	CHOLEST
3627	CXCL10	137,02	85,36	188,69	2,21	1,14	2,81E-19	3,90E-16	
1088	CEACAM8	583,42	777,45	389,40	0,50	-1,00	4,54E-19	5,88E-16	
671	BPI	954,54	1253,93	655,15	0,52	-0,94	1,37E-18	1,66E-15	
3002	GZMB	2759,21	1987,61	3530,81	1,78	0,83	2,31E-18	2,64E-15	
419	ART3	148,25	94,80	201,71	2,13	1,09	3,94E-18	4,25E-15	
10529	NEBL	146,58	91,30	201,85	2,21	1,14	4,16E-18	4,26E-15	
54094	C21orf15	381,10	519,20	243,00	0,47	-1,10	5,81E-18	5,65E-15	
80832	APOL4	84,32	51,76	116,88	2,26	1,18	1,05E-17	9,73E-15	
154664	ABCA13	292,58	392,10	193,06	0,49	-1,02	1,49E-17	1,32E-14	
3934	LCN2	1045,17	1358,09	732,25	0,54	-0,89	1,74E-17	1,47E-14	BMI
115362	GBP5	14422,47	10811,62	18033,31	1,67	0,74	2,14E-17	1,74E-14	
6557	SLC12A1	3200,27	2144,40	4256,14	1,98	0,99	2,64E-17	1,97E-14	
10501	SEMA6B	110,83	69,85	151,81	2,17	1,12	2,55E-17	1,97E-14	
4057	LTF	3796,89	4921,84	2671,95	0,54	-0,88	3,36E-17	2,42E-14	
820	CAMP	600,61	782,59	418,63	0,53	-0,90	5,49E-17	3,77E-14	
3119	HLA-DQB1	4843,38	3691,65	5995,10	1,62	0,70	5,62E-17	3,77E-14	CHOLEST
64478	CSMD1	50,11	70,46	29,77	0,42	-1,24	2,21E-16	1,43E-13	
2900	GRIK4	71,31	41,51	101,10	2,44	1,28	2,50E-16	1,57E-13	
4353	MPO	902,54	1170,11	634,97	0,54	-0,88	5,49E-16	3,34E-13	BMI, CHOLEST
1669	DEFA4	453,05	589,98	316,12	0,54	-0,90	1,30E-15	7,65E-13	
23026	MYO16	294,19	379,24	209,15	0,55	-0,86	3,24E-15	1,85E-12	
1281	COL3A1	5,09	0,51	9,68	18,86	4,24	1,27E-14	7,06E-12	CHOLEST
10321	CRISP3	274,44	360,34	188,54	0,52	-0,93	1,50E-14	8,13E-12	
3811	KIR3DL1	269,06	199,04	339,08	1,70	0,77	3,03E-14	1,59E-11	
116071	BATF2	305,99	227,10	384,87	1,69	0,76	4,00E-14	2,05E-11	
165530	CLEC4F	124,58	83,57	165,59	1,98	0,99	7,44E-14	3,71E-11	
566	AZU1	301,64	394,18	209,10	0,53	-0,91	9,46E-14	4,57E-11	
1991	ELANE	243,54	320,98	166,10	0,52	-0,95	9,63E-14	4,57E-11	
3804	KIR2DL3	161,19	111,53	210,86	1,89	0,92	1,22E-13	5,66E-11	
284581	LOC284581	180,20	230,18	130,21	0,57	-0,82	2,12E-13	9,59E-11	
1719	DHFR	1383,23	1131,85	1634,60	1,44	0,53	9,85E-13	4,36E-10	

4973	OLR1	89,24	120,90	57,58	0,48	-1,07	1,13E-12	4,88E-10	CHOLEST
1667	DEFA1	2160,26	2759,97	1560,56	0,57	-0,82	1,23E-12	4,99E-10	CHOLEST
728358	DEFA1B	2160,26	2759,97	1560,56	0,57	-0,82	1,23E-12	4,99E-10	
1668	DEFA3	2160,26	2759,97	1560,56	0,57	-0,82	1,23E-12	4,99E-10	CHOLEST
338785	KRT79	8,20	1,76	14,65	8,31	3,06	1,41E-12	5,58E-10	
8277	TKTL1	264,63	209,48	319,78	1,53	0,61	1,54E-12	6,00E-10	
5159	PDGFRB	307,12	228,51	385,74	1,69	0,76	1,62E-12	6,18E-10	
710	SERPING1	816,61	632,34	1000,89	1,58	0,66	1,86E-12	6,97E-10	
5266	PI3	940,57	1174,68	706,46	0,60	-0,73	1,97E-12	7,23E-10	
3809	KIR2DS4	238,22	186,96	289,47	1,55	0,63	2,36E-12	8,51E-10	
389396	GLYATL3	4,50	0,21	8,78	41,57	5,38	3,41E-12	1,21E-09	
5819	PVRL2	349,69	296,38	402,99	1,36	0,44	7,63E-12	2,65E-09	CHOLEST
93010	B3GNT7	145,42	105,23	185,62	1,76	0,82	1,27E-11	4,34E-09	
9289	GPR56	5845,55	4594,16	7096,95	1,54	0,63	1,34E-11	4,48E-09	
2999	GZMH	2449,28	1947,68	2950,88	1,52	0,60	1,94E-11	6,40E-09	
59352	LGR6	479,22	362,59	595,85	1,64	0,72	2,57E-11	8,34E-09	
4318	MMP9	4561,06	5592,53	3529,59	0,63	-0,66	2,67E-11	8,50E-09	BMI, CHOLEST
100463486	MTRNR2L8	186,52	145,67	227,38	1,56	0,64	3,28E-11	1,03E-08	
212	ALAS2	5232,68	4227,75	6237,61	1,48	0,56	3,89E-11	1,20E-08	
10398	MYL9	725,18	869,53	580,84	0,67	-0,58	3,99E-11	1,21E-08	
55007	FAM118A	2175,23	2611,56	1738,91	0,67	-0,59	4,25E-11	1,27E-08	
105	ADARB2	815,25	993,62	636,88	0,64	-0,64	5,09E-11	1,48E-08	
4680	CEACAM6	449,85	566,55	333,14	0,59	-0,77	5,09E-11	1,48E-08	
101059918	GOLGA8R	56,80	76,22	37,38	0,49	-1,03	6,21E-11	1,78E-08	
5473	PPBP	3174,53	3790,92	2558,13	0,67	-0,57	6,46E-11	1,82E-08	
9828	ARHGEF17	105,60	134,75	76,44	0,57	-0,82	1,43E-10	3,97E-08	
1240	CMKLR1	1409,52	1127,24	1691,80	1,50	0,59	1,78E-10	4,87E-08	
9047	SH2D2A	1505,31	1199,95	1810,67	1,51	0,59	2,47E-10	6,68E-08	
2633	GBP1	5081,66	4129,63	6033,68	1,46	0,55	2,99E-10	7,95E-08	
467	ATF3	73,88	52,41	95,36	1,82	0,86	4,57E-10	1,20E-07	CHOLEST
1690	COCH	208,40	160,42	256,38	1,60	0,68	5,20E-10	1,35E-07	
91977	MYOZ3	11,86	18,86	4,86	0,26	-1,96	6,23E-10	1,59E-07	
6037	RNASE3	213,96	275,55	152,36	0,55	-0,85	7,08E-10	1,79E-07	BMI
55966	AJAP1	203,48	250,73	156,23	0,62	-0,68	7,35E-10	1,83E-07	
5551	PRF1	17240,36	14073,54	20407,18	1,45	0,54	7,45E-10	1,83E-07	
1511	CTSG	144,86	186,38	103,34	0,55	-0,85	7,75E-10	1,88E-07	
3240	HP	217,19	272,47	161,91	0,59	-0,75	9,82E-10	2,36E-07	CHOLEST
383	ARG1	473,00	572,53	373,47	0,65	-0,62	1,02E-09	2,43E-07	
1844	DUSP2	884,12	726,53	1041,72	1,43	0,52	1,34E-09	3,15E-07	
5657	PRTN3	81,50	108,36	54,63	0,50	-0,99	1,64E-09	3,79E-07	
101927586	RP11-290F20.2	662,21	803,90	520,52	0,65	-0,63	1,94E-09	4,44E-07	
654341	TBC1D3G	2,88	0,18	5,58	31,55	4,98	2,32E-09	5,24E-07	
11098	PRSS23	661,12	526,99	795,25	1,51	0,59	2,49E-09	5,56E-07	
3812	KIR3DL2	207,87	164,49	251,25	1,53	0,61	3,21E-09	7,09E-07	
6861	SYT5	3,36	0,34	6,39	18,89	4,24	3,36E-09	7,33E-07	
399697	CTXN2	65,76	42,01	89,51	2,13	1,09	4,10E-09	8,86E-07	
6320	CLEC11A	145,07	180,14	109,99	0,61	-0,71	4,28E-09	9,14E-07	
7453	WARS	9293,85	7675,18	10912,52	1,42	0,51	4,46E-09	9,44E-07	
6351	CCL4	697,93	562,77	833,08	1,48	0,57	8,67E-09	1,81E-06	CHOLEST
100506071	RP11-829H16.3	166,92	130,00	203,83	1,57	0,65	9,49E-09	1,96E-06	
10316	NMUR1	904,41	729,78	1079,03	1,48	0,56	1,03E-08	2,11E-06	
152789	JAKMIP1	394,65	326,37	462,93	1,42	0,50	1,05E-08	2,12E-06	
2078	ERG	76,12	98,56	53,68	0,54	-0,88	1,11E-08	2,23E-06	BMI, CHOLEST
5273	SERPINB10	60,12	77,32	42,91	0,56	-0,85	1,29E-08	2,51E-06	
3486	IGFBP3	205,73	162,61	248,85	1,53	0,61	1,28E-08	2,51E-06	BMI, CHOLEST
136227	COL26A1	64,01	48,93	79,09	1,62	0,69	1,29E-08	2,51E-06	
284751	RP11-290F20.1	1440,14	1721,19	1159,10	0,67	-0,57	1,37E-08	2,63E-06	
1118	CHIT1	122,37	153,32	91,42	0,60	-0,75	1,69E-08	3,22E-06	
3690	ITGB3	1943,01	2302,91	1583,11	0,69	-0,54	1,72E-08	3,24E-06	CHOLEST
343413	FCRL6	1920,67	1569,09	2272,25	1,45	0,53	1,88E-08	3,51E-06	
26011	TENM4	51,02	37,10	64,94	1,75	0,81	2,05E-08	3,81E-06	
100996671	RP11-407A16.3	19,66	10,81	28,50	2,64	1,40	2,41E-08	4,42E-06	
114132	SIGLEC11	61,06	81,26	40,86	0,50	-0,99	2,43E-08	4,43E-06	

53637	S1PR5	2098,53	1727,70	2469,37	1,43	0,52	2,67E-08	4,81E-06	
91181	NUP210L	47,64	63,52	31,76	0,50	-1,00	3,46E-08	6,17E-06	
6512	SLC1A7	95,30	72,41	118,19	1,63	0,71	3,68E-08	6,51E-06	
3802	KIR2DL1	188,12	148,85	227,39	1,53	0,61	4,00E-08	7,01E-06	
54084	TSPEAR	360,50	433,02	287,97	0,67	-0,59	4,10E-08	7,11E-06	
3823	KLRC3	714,63	583,07	846,18	1,45	0,54	4,82E-08	8,30E-06	
100190986	LOC100190986	94,99	88,13	101,86	1,16	0,21	5,52E-08	9,41E-06	
9495	AKAP5	247,10	196,33	297,87	1,52	0,60	5,90E-08	9,97E-06	
9934	P2RY14	817,60	685,06	950,14	1,39	0,47	6,76E-08	1,13E-05	
6590	SLPI	336,96	409,30	264,61	0,65	-0,63	7,58E-08	1,26E-05	
5414	SEPT4	101,36	77,94	124,77	1,60	0,68	8,34E-08	1,37E-05	
8993	PGLYRP1	814,07	948,39	679,76	0,72	-0,48	1,14E-07	1,86E-05	
340024	SLC6A19	4,89	8,18	1,60	0,20	-2,35	1,22E-07	1,98E-05	
84680	ACCS	1344,47	1537,67	1151,27	0,75	-0,42	1,33E-07	2,14E-05	
116835	HSPA12B	20,81	12,28	29,35	2,39	1,26	1,46E-07	2,33E-05	
9891	NUAK1	43,68	29,25	58,11	1,99	0,99	1,54E-07	2,44E-05	
115361	GBP4	5351,52	4540,83	6162,21	1,36	0,44	1,65E-07	2,59E-05	
1277	COL1A1	12,82	6,94	18,71	2,70	1,43	1,73E-07	2,69E-05	
8671	SLC4A4	152,82	120,93	184,72	1,53	0,61	2,00E-07	3,08E-05	
1290	COL5A2	38,08	50,18	25,97	0,52	-0,95	2,05E-07	3,15E-05	
5627	PROS1	92,26	114,91	69,60	0,61	-0,72	2,22E-07	3,38E-05	
346171	ZFP57	61,44	44,96	77,92	1,73	0,79	3,11E-07	4,68E-05	
9510	ADAMTS1	223,02	179,82	266,23	1,48	0,57	3,53E-07	5,28E-05	
765	CA6	782,20	912,15	652,26	0,72	-0,48	3,86E-07	5,74E-05	
133396	IL31RA	86,26	67,44	105,08	1,56	0,64	4,06E-07	5,99E-05	
80310	PDGFD	385,33	317,63	453,03	1,43	0,51	4,64E-07	6,79E-05	
10562	OLFM4	499,76	594,33	405,20	0,68	-0,55	4,98E-07	7,22E-05	
6425	SFRP5	46,87	59,92	33,82	0,56	-0,83	5,31E-07	7,64E-05	
283726	FAM154B	98,60	69,49	127,71	1,84	0,88	5,34E-07	7,64E-05	
342184	FMN1	667,88	536,52	799,25	1,49	0,58	5,39E-07	7,65E-05	
9582	APOBEC3B	295,29	237,29	353,30	1,49	0,57	5,62E-07	7,91E-05	
30009	TBX21	2657,54	2231,66	3083,41	1,38	0,47	5,78E-07	8,08E-05	
283177	LOC283177	87,88	66,95	108,80	1,63	0,70	6,00E-07	8,34E-05	
57126	CD177	166,65	197,33	135,97	0,69	-0,54	6,19E-07	8,54E-05	
10578	GNLY	17422,45	14441,58	20403,32	1,41	0,50	6,38E-07	8,74E-05	
64105	CENPK	838,02	970,00	706,05	0,73	-0,46	6,45E-07	8,77E-05	
306	ANXA3	1310,91	1527,79	1094,02	0,72	-0,48	6,72E-07	9,07E-05	
4283	CXCL9	38,90	27,77	50,02	1,80	0,85	6,77E-07	9,08E-05	
1521	CTSW	5214,63	4426,88	6002,38	1,36	0,44	7,00E-07	9,32E-05	
23428	SLC7A8	199,76	243,03	156,50	0,64	-0,63	7,14E-07	9,44E-05	
7049	TGFBR3	2179,48	1834,87	2524,09	1,38	0,46	7,45E-07	9,79E-05	
81788	NUAK2	5820,68	4802,26	6839,10	1,42	0,51	1,10E-06	1,43E-04	
3048	HBG2	1475,79	1206,34	1745,23	1,45	0,53	1,14E-06	1,48E-04	CHOLEST
4818	NKG7	9266,13	7874,34	10657,93	1,35	0,44	1,16E-06	1,50E-04	
2149	F2R	1437,31	1220,36	1654,26	1,36	0,44	1,25E-06	1,60E-04	BMI, CHOLEST
654433	PAX8-AS1	1182,21	955,77	1408,65	1,47	0,56	1,34E-06	1,70E-04	
7849	PAX8	1453,81	1179,17	1728,44	1,47	0,55	1,61E-06	2,03E-04	
56101	PCDHGB5	199,59	159,90	239,28	1,50	0,58	1,62E-06	2,03E-04	
3852	KRT5	87,30	65,63	108,97	1,66	0,73	1,76E-06	2,20E-04	
9708	PCDHGA8	201,50	161,73	241,28	1,49	0,58	1,81E-06	2,24E-04	
83888	FGFBP2	2415,70	2039,13	2792,28	1,37	0,45	2,18E-06	2,69E-04	
3822	KLRC2	221,30	184,50	258,10	1,40	0,48	2,22E-06	2,72E-04	
8641	PCDHGB4	207,03	166,70	247,36	1,48	0,57	2,25E-06	2,73E-04	
728577	CNTNAP3B	28,67	19,16	38,18	1,99	0,99	2,34E-06	2,83E-04	
152404	IGSF11	22,36	30,44	14,28	0,47	-1,09	2,43E-06	2,92E-04	
56108	PCDHGA7	213,06	172,04	254,08	1,48	0,56	2,75E-06	3,28E-04	
5413	SEPT5	1225,83	1411,45	1040,21	0,74	-0,44	2,89E-06	3,43E-04	
23569	PADI4	5688,12	6576,01	4800,23	0,73	-0,45	3,13E-06	3,69E-04	
2201	FBN2	816,14	698,17	934,12	1,34	0,42	3,18E-06	3,72E-04	
79838	TMC5	279,67	325,77	233,57	0,72	-0,48	3,22E-06	3,75E-04	
441168	FAM26F	686,74	582,46	791,01	1,36	0,44	3,38E-06	3,91E-04	
51348	KLRF1	2586,50	2175,24	2997,76	1,38	0,46	3,48E-06	4,00E-04	
56107	PCDHGA9	188,03	151,41	224,65	1,48	0,57	3,57E-06	4,09E-04	

89944	GLB1L2	122,50	99,00	146,00	1,47	0,56	3,72E-06	4,23E-04	
51208	CLDN18	24,25	33,30	15,21	0,46	-1,13	3,78E-06	4,27E-04	
117157	SH2D1B	1301,34	1096,53	1506,14	1,37	0,46	4,59E-06	5,16E-04	
116173	CMTM5	155,77	183,72	127,83	0,70	-0,52	4,76E-06	5,28E-04	
56111	PCDHGA4	264,03	216,48	311,57	1,44	0,53	4,77E-06	5,28E-04	
56102	PCDHGB3	244,82	200,00	289,64	1,45	0,53	4,78E-06	5,28E-04	
56110	PCDHGA5	251,86	205,99	297,74	1,45	0,53	4,90E-06	5,38E-04	
56113	PCDHGA2	267,51	219,51	315,52	1,44	0,52	4,94E-06	5,40E-04	
56112	PCDHGA3	265,48	217,87	313,09	1,44	0,52	5,08E-06	5,52E-04	
125875	CLDND2	298,73	246,91	350,55	1,42	0,51	5,14E-06	5,52E-04	
56103	PCDHGB2	262,04	214,98	309,09	1,44	0,52	5,13E-06	5,52E-04	
56104	PCDHGB1	264,81	217,40	312,21	1,44	0,52	5,23E-06	5,59E-04	
6696	SPP1	52,72	66,31	39,13	0,59	-0,76	5,65E-06	6,01E-04	CHOLEST
56100	PCDHGB6	183,72	148,55	218,90	1,47	0,56	5,75E-06	6,08E-04	
3821	KLRC1	329,43	274,74	384,13	1,40	0,48	6,08E-06	6,39E-04	
56109	PCDHGA6	233,79	191,08	276,49	1,45	0,53	6,31E-06	6,60E-04	
932	MS4A3	581,71	680,28	483,13	0,71	-0,49	6,51E-06	6,77E-04	
5097	PCDH1	115,21	92,19	138,23	1,50	0,58	7,12E-06	7,36E-04	
3123	HLA-DRB1	8296,17	7381,43	9210,91	1,25	0,32	7,48E-06	7,70E-04	CHOLEST
222389	BEND7	104,23	125,53	82,92	0,66	-0,60	7,66E-06	7,84E-04	
2944	GSTM1	306,29	359,81	252,78	0,70	-0,51	8,04E-06	8,17E-04	CHOLEST
10699	CORIN	170,15	140,88	199,42	1,42	0,50	8,07E-06	8,17E-04	
56114	PCDHGA1	271,90	224,46	319,35	1,42	0,51	8,47E-06	8,54E-04	
6606	SMN1	169,89	203,05	136,73	0,67	-0,57	9,18E-06	9,15E-04	
6607	SMN2	169,89	203,05	136,73	0,67	-0,57	9,18E-06	9,15E-04	
64284	RAB17	6,63	2,68	10,57	3,94	1,98	1,01E-05	1,00E-03	
5149	PDE6H	18,23	11,10	25,35	2,28	1,19	1,03E-05	1,02E-03	
1824	DSC2	1476,69	1720,58	1232,80	0,72	-0,48	1,19E-05	1,17E-03	
127435	PODN	78,89	61,74	96,03	1,56	0,64	1,23E-05	1,20E-03	
10417	SPON2	4867,27	4170,77	5563,77	1,33	0,42	1,32E-05	1,28E-03	
8218	CLTCL1	478,56	562,15	394,97	0,70	-0,51	1,44E-05	1,39E-03	
5730	PTGDS	779,60	667,82	891,37	1,33	0,42	1,44E-05	1,39E-03	
79971	WLS	2486,88	2832,27	2141,49	0,76	-0,40	1,53E-05	1,47E-03	
55384	MEG3	76,34	54,62	98,06	1,80	0,84	1,60E-05	1,52E-03	
66000	TMEM108	134,76	157,73	111,78	0,71	-0,50	1,62E-05	1,53E-03	
23336	SYNM	330,20	283,91	376,49	1,33	0,41	1,72E-05	1,62E-03	
57595	PDZD4	1576,13	1355,88	1796,37	1,32	0,41	1,78E-05	1,67E-03	
1308	COL17A1	59,39	74,38	44,40	0,60	-0,74	1,86E-05	1,74E-03	
401124	DTHD1	503,22	430,15	576,28	1,34	0,42	1,92E-05	1,79E-03	
3824	KLRD1	2799,67	2427,52	3171,82	1,31	0,39	1,99E-05	1,84E-03	
100506084	ARL17B	270,15	222,09	318,20	1,43	0,52	2,03E-05	1,87E-03	
81563	C1orf21	643,50	554,34	732,67	1,32	0,40	2,07E-05	1,87E-03	
4684	NCAM1	795,37	689,07	901,67	1,31	0,39	2,07E-05	1,87E-03	
51326	ARL17A	270,26	222,20	318,31	1,43	0,52	2,04E-05	1,87E-03	
56603	CYP26B1	49,37	35,85	62,89	1,75	0,81	2,06E-05	1,87E-03	
946	SIGLEC6	138,84	113,81	163,87	1,44	0,53	2,18E-05	1,95E-03	
3001	GZMA	2817,59	2440,40	3194,78	1,31	0,39	2,17E-05	1,95E-03	
4929	NR4A2	115,76	96,01	135,51	1,41	0,50	2,30E-05	2,05E-03	
57419	SLC24A3	226,90	263,71	190,08	0,72	-0,47	2,34E-05	2,08E-03	
30848	CTAG2	1,66	0,22	3,10	14,33	3,84	2,43E-05	2,15E-03	
90011	KIR3DX1	62,01	47,60	76,41	1,61	0,68	2,47E-05	2,17E-03	
353299	RGSL1	17,88	24,67	11,09	0,45	-1,15	2,48E-05	2,18E-03	
266629	SEC14L3	51,90	64,31	39,50	0,61	-0,70	2,52E-05	2,20E-03	
666	BOK	170,14	143,15	197,12	1,38	0,46	2,58E-05	2,24E-03	
57823	SLAMF7	3132,65	2743,71	3521,59	1,28	0,36	2,72E-05	2,31E-03	
7052	TGM2	115,00	97,54	132,46	1,36	0,44	2,69E-05	2,31E-03	
2815	GP9	263,23	302,38	224,08	0,74	-0,43	2,69E-05	2,31E-03	
27242	TNFRSF21	203,85	169,20	238,50	1,41	0,50	2,71E-05	2,31E-03	
4603	MYBL1	3089,16	2685,09	3493,22	1,30	0,38	2,71E-05	2,31E-03	
200132	TCTEX1D1	29,88	38,07	21,68	0,57	-0,81	2,82E-05	2,39E-03	
3674	ITGA2B	1901,65	2153,26	1650,03	0,77	-0,38	3,04E-05	2,56E-03	
4481	MSR1	211,57	191,40	231,74	1,21	0,28	3,05E-05	2,56E-03	
150165	XKR3	81,17	102,78	59,56	0,58	-0,79	3,13E-05	2,61E-03	

28959	TMEM176B	1742,43	1894,04	1590,82	0,84	-0,25	3,18E-05	2,64E-03	
41	ASIC1	58,12	70,93	45,31	0,64	-0,65	3,25E-05	2,69E-03	
4914	NTRK1	216,86	182,08	251,65	1,38	0,47	3,29E-05	2,71E-03	BMI
2258	FGF13	61,34	74,28	48,40	0,65	-0,62	3,30E-05	2,71E-03	
440823	MIAT	5852,05	5089,94	6614,16	1,30	0,38	3,37E-05	2,75E-03	
56106	PCDHGA10	166,37	136,85	195,89	1,43	0,52	3,66E-05	2,98E-03	
27239	GPR162	1313,53	1540,89	1086,17	0,70	-0,50	3,93E-05	3,19E-03	
713	C1QB	65,66	53,16	78,16	1,47	0,56	4,02E-05	3,24E-03	
51148	CERCAM	81,35	63,92	98,78	1,55	0,63	4,06E-05	3,27E-03	
9231	DLG5	460,65	388,67	532,63	1,37	0,45	4,16E-05	3,33E-03	
283358	B4GALNT3	213,00	185,43	240,58	1,30	0,38	4,41E-05	3,52E-03	
23601	CLEC5A	402,64	468,45	336,84	0,72	-0,48	4,85E-05	3,85E-03	
1908	EDN3	16,40	22,79	10,02	0,44	-1,19	5,15E-05	4,07E-03	
1053	CEBPE	349,01	401,07	296,95	0,74	-0,43	5,35E-05	4,21E-03	
9914	ATP2C2	134,18	158,67	109,69	0,69	-0,53	5,79E-05	4,54E-03	
55365	TMEM176A	626,05	679,11	573,00	0,84	-0,25	5,93E-05	4,63E-03	
285852	TREML4	454,35	392,31	516,38	1,32	0,40	6,09E-05	4,74E-03	
2791	GNG11	802,90	901,24	704,56	0,78	-0,36	6,12E-05	4,74E-03	
1663	DDX11	1069,72	1170,44	968,99	0,83	-0,27	6,27E-05	4,84E-03	
2359	FPR3	194,12	165,43	222,81	1,35	0,43	6,55E-05	5,04E-03	
8082	SSPN	146,29	122,57	170,01	1,39	0,47	6,59E-05	5,05E-03	
5196	PF4	1268,74	1413,37	1124,12	0,80	-0,33	6,67E-05	5,09E-03	BMI
100534611	TM4SF19-TCTEX1D2	236,64	200,44	272,84	1,36	0,44	6,71E-05	5,10E-03	
3120	HLA-DQB2	48,40	36,34	60,46	1,66	0,73	6,77E-05	5,12E-03	
1667	DEFA1	3,42	5,79	1,04	0,18	-2,47	6,86E-05	5,15E-03	CHOLEST
728358	DEFA1B	3,42	5,79	1,04	0,18	-2,47	6,86E-05	5,15E-03	
1634	DCN	6,18	3,18	9,19	2,89	1,53	6,89E-05	5,15E-03	
64167	ERAP2	5615,76	4989,48	6242,05	1,25	0,32	6,95E-05	5,18E-03	
6753	SSTR3	365,31	414,79	315,83	0,76	-0,39	7,20E-05	5,34E-03	
116534	MRGPRE	23,01	29,92	16,09	0,54	-0,89	7,83E-05	5,78E-03	
56099	PCDHGB7	154,81	128,44	181,19	1,41	0,50	7,85E-05	5,78E-03	
387509	GPR153	127,70	106,74	148,66	1,39	0,48	7,90E-05	5,80E-03	
5098	PCDHGC3	132,55	108,98	156,12	1,43	0,52	8,06E-05	5,89E-03	
89872	AQP10	76,77	92,09	61,44	0,67	-0,58	8,27E-05	6,02E-03	
9398	CD101	1739,15	1948,97	1529,34	0,78	-0,35	8,39E-05	6,09E-03	
94025	MUC16	15,23	10,16	20,30	2,00	1,00	8,50E-05	6,14E-03	CHOLEST
26025	PCDHGA12	140,52	116,12	164,92	1,42	0,51	8,60E-05	6,17E-03	
23500	DAAM2	310,78	265,80	355,75	1,34	0,42	8,60E-05	6,17E-03	
1832	DSP	172,18	134,73	209,62	1,56	0,64	8,95E-05	6,40E-03	
116448	OLIG1	974,59	1089,16	860,03	0,79	-0,34	9,24E-05	6,58E-03	
84525	HOPX	970,87	838,02	1103,72	1,32	0,40	9,95E-05	7,04E-03	
54796	BNC2	128,09	107,54	148,64	1,38	0,47	9,95E-05	7,04E-03	
9022	CLIC3	489,84	430,05	549,62	1,28	0,35	1,01E-04	7,08E-03	
3805	KIR2DL4	66,52	54,54	78,49	1,44	0,53	1,03E-04	7,20E-03	
4052	LTBP1	239,13	277,31	200,94	0,72	-0,46	1,04E-04	7,31E-03	
54836	BSPRY	32,34	23,81	40,87	1,72	0,78	1,07E-04	7,47E-03	
79623	GALNT14	172,67	196,79	148,55	0,75	-0,41	1,08E-04	7,47E-03	
219537	SMTNL1	276,11	241,40	310,82	1,29	0,36	1,10E-04	7,58E-03	
90102	PHLDB2	384,94	331,81	438,07	1,32	0,40	1,10E-04	7,58E-03	
152687	ZNF595	392,22	335,18	449,26	1,34	0,42	1,15E-04	7,88E-03	
554226	ANKRD30BL	268,15	304,30	232,00	0,76	-0,39	1,19E-04	8,12E-03	
631	BFSP1	124,73	103,93	145,54	1,40	0,49	1,19E-04	8,12E-03	
56105	PCDHGA11	149,89	124,77	175,02	1,40	0,49	1,21E-04	8,19E-03	
10231	RCAN2	31,55	24,61	38,49	1,56	0,65	1,21E-04	8,19E-03	
8787	RGS9	454,40	395,59	513,22	1,30	0,38	1,22E-04	8,22E-03	
2946	GSTM2	1211,84	1354,85	1068,83	0,79	-0,34	1,25E-04	8,41E-03	
9437	NCR1	425,46	365,43	485,50	1,33	0,41	1,25E-04	8,41E-03	
23547	LILRA4	489,06	421,31	556,80	1,32	0,40	1,27E-04	8,49E-03	
192683	SCAMP5	294,54	251,63	337,46	1,34	0,42	1,28E-04	8,51E-03	
100506159	LOC100506159	103,74	124,83	82,64	0,66	-0,59	1,33E-04	8,86E-03	
55020	TTC38	1678,86	1471,58	1886,14	1,28	0,36	1,45E-04	9,58E-03	
249	ALPL	7822,57	8734,78	6910,35	0,79	-0,34	1,47E-04	9,69E-03	CHOLEST
126326	GIPC3	223,25	253,15	193,35	0,76	-0,39	1,48E-04	9,69E-03	

6915	TBXA2R	141,25	164,03	118,47	0,72	-0,47	1,48E-04	9,69E-03	
60675	PROK2	5756,30	6396,31	5116,29	0,80	-0,32	1,48E-04	9,69E-03	
114884	OSBPL10	413,24	352,05	474,43	1,35	0,43	1,49E-04	9,72E-03	CHOLEST
9254	CACNA2D2	793,35	694,79	891,91	1,28	0,36	1,53E-04	9,89E-03	
60489	APOBEC3G	2697,98	2375,05	3020,90	1,27	0,35	1,54E-04	9,94E-03	

Headers of the Table	
geneID	Gene Identification
Gene Symbol	Official Symbol
Base Mean	Mean normalized counts, averaged over all samples from both conditions
Base Mean Weight Gain	Mean normalized counts from condition A
Base Mean No Weight Gain	Mean normalized counts from condition B
Fold Change	Fold change from condition A to B (B/A)
Log2 Fold Change	The logarithm, to basis 2, of the fold change
Pval	P value for the statistical significance of this change
Padj	P value adjusted for multiple testing with the Benjamini-Hochberg procedure, which controls false discovery rate
GeneRif Annotation	Genes including the strings “BMI” or “cholest” in their GeneRIF definition
